# Characterizing Tractability of Simple Well-Designed Pattern Trees with Projection

**DOI:** 10.1007/s00224-020-10002-z

**Published:** 2020-09-10

**Authors:** Stefan Mengel, Sebastian Skritek

**Affiliations:** 1 CRIL, CNRS & Université d’Artois, Lens, France; 2 Faculty of Informatics, TU Wien, Vienna, Austria

**Keywords:** SPARQL, Well-designed pattern trees, Query evaluation, FPT, Characterizing tractable classes

## Abstract

We study the complexity of evaluating well-designed pattern trees, a query language extending conjunctive queries with the possibility to define parts of the query to be optional. This possibility of optional parts is important for obtaining meaningful results over incomplete data sources as it is common in semantic web settings. Recently, a structural characterization of the classes of well-designed pattern trees that can be evaluated in polynomial time was shown. However, projection—a central feature of many query languages—was not considered in this study. We work towards closing this gap by giving a characterization of all tractable classes of simple well-designed pattern trees with projection (under some common complexity theoretic assumptions). Since well-designed pattern trees correspond to the fragment of well-designed {AND, OPTIONAL}-SPARQL queries this gives a complete description of the tractable classes of queries with projections in this fragment that can be characterized by the underlying graph structures of the queries. For non-simple pattern trees the tractability criteria for simple pattern trees do not capture all tractable classes. We thus extend the characterization for the non-simple case in order to capture some additional tractable cases.

## Introduction

Well-designed pattern trees (wdPTs) are a query formalism well-suited to deal with the ever increasing amount of incomplete data. Well-designed pattern trees over SPARQL triple patterns are equivalent to the class of well-designed {AND, OPTIONAL}-SPARQL queries, see Pérez et al. [21], and were in fact originally introduced as a formalism to more easily study SPARQL queries. By replacing triple patterns with relational atoms, wdPTs can also be seen as an extension of Conjunctive Queries (CQs): a wdPT is a rooted tree where each node represents a conjunction of atoms, and the tree structure represents a nesting of optional matching. The idea is to start evaluating the CQ at the root and to iteratively extend the retrieved results as much as possible by the results of the CQs in the other nodes. This allows wdPTs to return partial answers in cases where mapping the complete query into the database is impossible—unlike CQs which in such a situation return no answer.

Well-designed pattern trees and the corresponding SPARQL fragment represent an important class of SPARQL queries and have been studied intensively within the last decade, see Pérez et al. [21], Letelier et al. [17], Arenas and Pérez [1], Pichler and Skritek [23], Picalausa and Vansummeren [22], Kostylev et al. [15], Barceló et al. [3], Arenas et al. [2], Romero [24]. Thus, many properties of and problems related to these queries are now well understood. For example, the evaluation problem for wdPTs (i.e., given a wdPT, a database and a mapping, is this mapping an answer to the wdPT over the database?) is coNP-complete for projection free wdPTs [21] and Σ2*P*-complete in the presence of projection [17]. However, certain tractable classes of wdPTs have been identified [3]. The main idea there is to extend known tractability conditions for CQs to wdPTs. However, the question of characterizing exactly the classes of wdPTs for which tractable query evaluation is possible—and thus the question of how suitable the approach of extending tractability conditions of CQs to wdPTs is for describing the space of tractable classes of wdPTs—has been largely ignored. Only very recently, this question was addressed for wdPTs without projection, and a characterization of the classes for which query evaluation is in PTIME was given by Romero [24]. Notably, as also observed for Boolean Conjunctive Queries by Grohe et al. [13] and Grohe [12], for wdPTs without projection these classes coincide with the ones for which evaluation is in FPT.

However, Romero [24] does not consider projection, an essential and central feature of query languages. Thus, the question “What are all tractable classes of wdPTs with projection?” remains open. We work towards closing this gap.

One observation consistently made in all aforementioned work on wdPTs is that problems become much more complex once projection is included. This is true for the computational complexity of the problems (e.g., as mentioned, for the evaluation problem it increases from coNP- to Σ2*P*-completeness; for classical query containment, the NP-complete problem becomes even undecidable [23]) as well as for establishing these results.

This is because of the particular semantics of well-designed SPARQL with projection. For wdPTs *without* projection, given some database, the set of answers consists of all variable mappings such that there exists a subtree of the wdPT satisfying the following conditions: first, it must contain the root node of the tree. Second, the set of variables occurring in the subtree must be the same as the domain of the mapping. Third, the mapping must map each atom in the subtree into the database, and fourth, no extension of the mapping is allowed to map all atoms of any node outside the subtree that is a child node of a node in the subtree into the database. This is illustrated by the following example (a precise definition is given in Section 2).

### *Example 1*

**Fig. 1:**
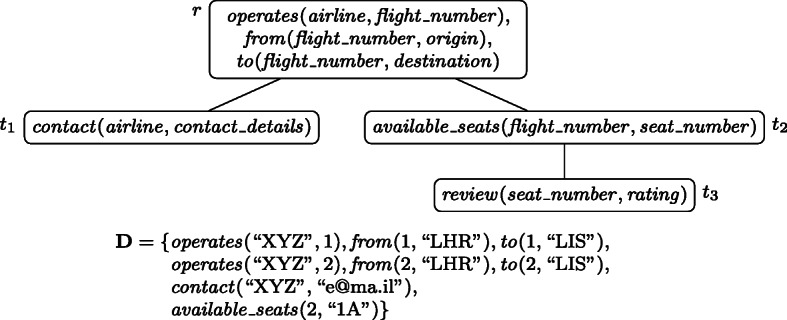
The wdPT *p* and database **D** from Example 1

Fig. 1 shows a wdPT *p* with four nodes *r*, *t*_1_, *t*_2_, and *t*_3_ where *r* is the root node.

At its root node, the query is looking for information on flights: the airline operating the flight, the flight number, origin and destination. This information shall be extended by some contact information (*t*_1_), and information on available seats on the flight (*t*_2_) in case any of this information is available. If, in addition to the information on available seats also some ratings of the free seats are available (*t*_3_), these shall be returned as well. Observe that the extensions to *t*_1_ and *t*_2_ are independent of each other. An equivalent SPARQL query (replacing relational atoms by triple patterns, and abbreviating variable and predicate names) would be

{{?airl operates ?fn . ?fn from ?origin . ?fn to ?dest} OPTIONAL {?airl contact ?cd} } OPTIONAL { {?fn avail_seats ?sn} OPTIONAL {?sn reviews ?r} }

For the database instance **D** also shown in the figure, the mapping *μ* defined as *μ*(*airline*) = “XYZ”, *μ*(*flight*_*number*) = 1, *μ*(*origin*) = “LHR”, *μ*(*destination*) = “LIS”, and *μ*(*contact*_*details*) = “e@ma.il” is an answer to *p* over **D**. This is because of the subtree of *p* consisting of the nodes *r* and *t*_1_. It can be checked that it satisfies all four conditions mentioned earlier. For the fourth condition, just observe that there exists no extension of *μ* that maps *available*_*seats*(*flight*_*number*, *seat*_*number*) into **D**. Because of the fourth condition, the mapping *ν* with *ν*(*airline*) = “XYZ”, *ν*(*flight*_*number*) = 2, *ν*(*origin*) = “LHR”, *ν*(*destination*) = “LIS”, and *μ*(*contact*_*details*) = “e@ma.il” is no solution, because this mapping can be extended by *ν*(*seat*_*number*) = “1A” in a way that maps *available*_*seats*(*flight*_*number*, *seat*_*number*) also into **D**.

Without projection, the only hard part in deciding whether some mapping is a solution is to check for the existence of an extension. This requires a homomorphism test which is well known to be an NP-hard problem. However, for wdPTs *with* projection, a mapping is a solution if there exists an extension of this mapping to some subset of the existential variables in the tree, such that the extended mapping is a solution to the wdPT considered without projection.

### *Example 2*

Consider the wdPT from Example 1, but now assume that the variables *flight*_*number* and *airline* are not part of the output but projected away. Then the mapping *μ* with *μ*(*origin*) = “LHR”,*μ*(*destination*) = “LIS”, and *μ*(*contact*_*details*) = “e@ma.il” is a solution because of the extension *μ*(*flight*_*number*) = 1 and *μ*(*airline*) = “XYZ”. Observe that the extension *μ*(*airline*) = “XYZ” and *μ*(*flight*_*number*) = 2 does not witness *μ* to be a solution, since, as already discussed before, this mapping is not maximal. As a result both, *μ* and its extension *ν* with *ν*(*seat*_*number*) = “1A” (and otherwise defined like *μ*) are solutions in this case.

As a consequence, besides testing some mapping for maximality, as a second source of hardness, different mappings on the existential variables have to be taken into account. In addition to the increased complexity of the evaluation problem, this also has the effect that the classes of wdPTs with projection for which query evaluation is in PTIME and in FPT no longer coincide, as observed by Kröll et al. [16]. Thus, in this setting, the choice of the tractability notion makes a difference when describing all tractable classes.

We choose to study the complexity of query evaluation in the model of parameterized complexity where, as usual, we take the size of the query as the parameter. As already argued by Papadimitriou and Yannakakis [20], this model allows for a more fine-grained analysis than the classical perspectives of data- and query complexity. In parameterized complexity, query answering is considered tractable, formally in FPT, if, after a preprocessing that only depends on the query, the actual evaluation can be done in polynomial time [10, 11]. This allows for potentially costly preprocessing on the generally small query while the dependency on the generally far bigger database is polynomial for an exponent independent of the query. Parameterized complexity has found many applications in the complexity of query evaluation problems, see, e.g., Grohe et al. [13], Grohe [12], Marx [18], Chen [4], Romero [24].

In our efforts to better understand the tractability frontier for wdPTs, we provide a complete characterization of the tractable classes of *simple* wdPTs, i.e., wdPTs where no two atoms share the same relation symbol. Because of the relationship between wdPTs and well-designed {AND, OPTIONAL}-SPARQL queries, this immediately gives a complete description of the tractable classes of well-designed {AND, OPTIONAL}-SPARQL queries with projection that can be characterized by only considering the graph structures of the queries, similar, e.g., to the work of Grohe et al. [13] and Chen [4]. We note that the results showing the existence of classes of wdPTs for which the evaluation problem is NP-hard but in FPT can be easily extended to simple wdPTs.

Our tractability criteria are not restricted to simple wdPTs. In fact, the same tractability criteria can also directly be applied to give tractable classes of non-simple wdPTs, i.e., such in which the same relational symbol may appear several times. However, in this case, there are classes of queries that do not satisfy our tractability criteria for simple queries and are still tractable. This shows that the restriction to simple wdPTs is crucial for the lower bounds. To extend the applicability of our techniques in the case of non-simple queries, we generalize our criteria by incorporating the notion of *cores* into well-designed pattern trees as it was done in the projection free case by Romero [24] and for conjunctive queries by Dalmau et al. [7]. While this allows us to show tractability for more classes of wdPTs, we do not achieve a full dichotomy in this setting.

### Summary of Results and Organization of the Paper

We study the following decision problem: Given a wdPT, a database, and a mapping, is the mapping a solution of the wdPT over the database? This is the standard formulation of the evaluation problem usually studied, cf. Letelier et al. [17], Kaminski and Kostylev [14], Romero [24], Barceló et al. [3]. It reveals the influence of the optional query parts on the evaluation problem, which is lost, e.g., when considering Boolean queries. Instead of just SPARQL triple patterns, we consider the more general case of wdPTs with arbitrary relational atoms where we always assume that the classes of queries we consider have bounded arity. Our main result is a characterization of the classes of simple wdPTs with projection that allow fixed-parameter tractable query evaluation.

After some preliminaries in Section 2, we define two tractability conditions in Section 3. By comparing these conditions with the tractability criterion given by Romero [24] for the projection free case, we discuss how they describe the additional complexity introduced by projection. Note that some of the conditions provided here have precursors in Barceló et al. [3] and Kröll et al. [16] that had to be carefully refined to provide a fine-grained complexity analysis.

In Section 4 we prove that the two tractability conditions imply FPT membership of the evaluation problem by presenting an algorithm that exploits these conditions.

In Section 5 we then show that both tractability conditions are indeed necessary for a class of simple wdPTs to be tractable. That is, we show that if either of them is not satisfied by a class of wdPTs, the evaluation problem for this class is either W[1]- or coW[1]-hard.

In Section 6 we show how to extend our tractability criteria for the non-simple case by incorporating the notion of cores and show how this allows us to capture more tractable classes. Besides a generalization of the tractability criteria from Section 3, we also introduce a generalization of the homomorphism problem, and completely characterize its tractable classes.

In Section 7, we discuss our results and potential extensions to conclude the paper.

This article is an extended version of the conference paper [19]. The main additional contribution compared to the conference version consists of Section 6, which is completely new. In addition, several minor improvements have been made throughout the article, including the extension of existing and addition of new examples.

## Preliminaries

### Basics

Let ${\mathcal Const}$ and ${\mathcal Var}$ be two disjoint countable infinite sets of constants and variables, respectively. A *relational schema* *σ* is a set $\{R_{1}, \dots , R_{n}\}$ of relation symbols *R*_*i*_, each having an assigned arity *r*_*i*_ ≥ 0. A *relational atom* *R*_*i*_(**v**) over *σ* consists of a relation symbol *R*_*i*_ ∈ *σ* and a tuple $\mathbf {v} \in ({\mathcal Const} \cup {\mathcal Var})^{r_{i}}$. For an atom *τ* = *R*_*i*_(**v**), let dom(*τ*) denote the set of variables and constants occurring in **v**. This extends to sets $\mathbf {R} = \{\tau _{1}, \dots , \tau _{m}\}$ of atoms as dom$(\mathbf {R}) = \bigcup _{i=1}^{m} \mathsf {dom}(\tau _{i})$. Furthermore, var$(\tau ) = \mathsf {dom}(\tau ) \cap {\mathcal Var}$ and var$(\mathbf {R}) = \mathsf {dom}(\mathbf {R}) \cap {\mathcal Var}$. Observe that, by slight abuse of notation, we use the operators ∪,∩,∖ also between sets $\mathcal {V}$ and tuples **v** of variables and constants. For example, var$(\tau ) = \mathbf {v} \cap {\mathcal Var}$. We call a set of atoms *simple* if no relation symbol appears more than once in it.

Similarly, for a mapping *μ* we denote with dom(*μ*) the set of elements on which *μ* is defined. For a mapping *μ* and a set $\mathcal {V} \subseteq {\mathcal Var}$, we use $\mu |_{\mathcal {V}}$ to describe the restriction of *μ* to the variables in dom$(\mu ) \cap \mathcal {V}$. We say that a mapping *μ* is an *extension* of a mapping *ν* if *μ*|_dom(*ν*)_ = *ν*, and that two mappings are *compatible* if they agree on the shared variables.

For a set **A** of atoms and a set $\mathcal {A} \subseteq \mathsf {dom}(\mathbf {A})$, we write $\mathbf {A} \setminus \mathcal {A}$ to denote the restriction of **A** to dom$(\mathbf {A}) \setminus \mathcal {A}$. That is, we substitute every atom *R*(**v**) ∈**A** by an atom $R^{s}(\mathbf {v}^{\prime })$, where $\mathbf {v}^{\prime }$ is obtained from **v** by removing elements of $\mathcal {A}$, and *s* is the list of the removed positions and their values.

A *database* **D** over *σ* is a finite set of atoms over *σ* with var(**D**) = *∅*. For a database **D** and relation symbol *R* we denote by *R*^**D**^ the set of all atoms in **D** with relation symbol *R*.

### Homomorphisms and Cores

A homomorphism *h* between two sets **A** and **B** of atoms over *σ* is a mapping $h \colon \mathsf {dom}(\mathbf {A}) \rightarrow \mathsf {dom}(\mathbf {B})$ such that for all atoms *R*(**v**) ∈**A** we have *R*(*h*(**v**)) ∈**B**, and such that *h*(*x*)≠*x* is only allowed if *x* ∈*var*(**A**) (throughout the article, when defining homomorphisms we therefore only state the mapping on var(**A**), and assume the extension to constants via the identity mapping to be implicit). We write $h\colon \mathbf {A} \rightarrow \mathbf {B}$ to denote a homomorphism *h* from **A** to **B**.

Let **A** be a set of atoms. A minimal subset $\mathbf {A}^{\prime } \subseteq \mathbf {A}$ such that there is a homomorphism $\mathbf {A}\rightarrow \mathbf {A}^{\prime }$ is called a *core* of **A**. We recall that all cores of **A** are unique up to isomorphism and thus speak of *the* core of **A** which we denote by core(**A**).

### Conjunctive Queries

We write conjunctive queries (short CQs) *q* as $\textit {Ans}(\mathbf {x}) \leftarrow \mathbf {B}$, where the body $\mathbf {B} = \{R_{1}(\mathbf {v}_{1}), \dots , R_{m}(\mathbf {v}_{m})\}$ is a set of atoms and **x** are the *free variables*. A Boolean CQ (BCQ) is a CQ with no free variables. We define var(*q*) = *var*(**B**). The existential variables are implicitly given by var(**B**) ∖**x**. The result *q*(**D**) of *q* over a database **D** is the set of tuples $\{h(\mathbf {x}) \mid h \colon \mathbf {B} \rightarrow \mathbf {D}\}$, i.e., every homomorphism mapping the body of the query into the database is projected onto the values assigned to the free variables.

### Graphs

We consider only undirected, simple graphs *G* = (*V*, *E*) with standard notations. We sometimes write *t* ∈ *G* to refer to a node *t* ∈ *V* (*G*). A graph *G*_2_ is a subgraph of a graph *G*_1_ if $V(G_{2}) \subseteq V(G_{1})$ and $E(G_{2}) \subseteq E(G_{1})$. For a graph *G* = (*V*, *E*) and a set $V^{\prime } \subseteq V$, the *induced graph* $G[V^{\prime }]$ is the subgraph $G[V^{\prime }] = (V^{\prime }, \{ \{v_{i},v_{j}\} \in E \mid v_{i},v_{j} \in V^{\prime }\})$. A tree is a connected, acyclic graph. A subtree is a connected, acyclic subgraph. A *rooted tree* *T* is a tree with one node *r* ∈ *T* marked as its root. Given two nodes $t, \hat t \in T$, we say that $\hat t$ is an ancestor of *t* if $\hat t$ lies on the path from *r* to *t*. Likewise, $\hat t$ is the parent node of *t* (and *t* is a child of $\hat t$) if $\hat t$ is an ancestor of *t* and $\{t, \hat t\} \in E(T)$. For a subtree $T^{\prime }$ of *T*, a node *t* ∈ *T* is a child of $T^{\prime }$ if $t \notin T^{\prime }$ and $\hat t \in T^{\prime }$ for the parent node $\hat t$ of *t*. We write $ch(T^{\prime })$ for the set of all children of $T^{\prime }$. For a node *t* ∈ *T* the set of nodes on the path from the root *r* to the parent node of *t* is denoted by branch(*t*). Moreover, cbranch(*t*) = *branch*(*t*) ∪{*t*}.

For a set **A** of atoms, the *Gaifman graph* of **A** is the graph *G* = (*V*, *E*) with *V* = {*v*_*i*_∣*v*_*i*_ ∈*var*(**A**)} and *E* contains an edge {*v*_*i*_, *v*_*j*_} if *v*_*i*_ and *v*_*j*_ occur together in some atom in **A**.

### Tree Decompositions and Treewidth

A *tree decomposition* of a graph *G* = (*V*, *E*) is a pair (*T*, *ν*), where *T* is a tree and *ν* : *V* (*T*) → 2^*V*^, that satisfies the following:

1. For each *u* ∈ *V* the set {*s* ∈ *V* (*T*)∣*u* ∈ *ν*(*s*)} is a connected subset of *V* (*T*), and
2. each edge of *E* is contained in at least one of the sets *ν*(*s*), for *s* ∈ *V* (*T*).

The *width* of (*T*, *ν*) is $(\max \limits {\{|\nu (s)| \mid s \in V(T)\}}) - 1$. The *treewidth* of *G* is the minimum width of its tree decompositions.

The treewidth of a set of atoms is the treewidth of its Gaifman graph.

### Well-Designed Pattern Trees (wdPTs)

A *pattern tree* (short: PT) *p* over a relational schema *σ* is a tuple $(T, \lambda , \mathcal {X})$ where *T* is a rooted tree and *λ* maps each node *t* ∈ *T* to a set of relational atoms over *σ*. We may write $((T,r), \lambda , \mathcal {X})$ to emphasize that *r* is the root node of *T*. The set $\mathcal {X}$ of variables denotes the *free variables* of the PT. For a PT $(T, \lambda , \mathcal {X})$ and a subtree $T^{\prime }$ of *T*, let $\lambda (T^{\prime }) = \bigcup _{t \in V(T^{\prime })}\lambda (t)$. We may write var(*t*) instead of var(*λ*(*t*)), and var$(T^{\prime })$ instead of var$(\lambda (T^{\prime }))$. We further define fvar$(t) = \mathsf {var}(t) \cap \mathcal {X}$ as the free variables in *t*. Again this definition extends naturally to subtrees $T^{\prime }$ of *T*. We call a PT $(T, \lambda , \mathcal {X})$ *projection free* if $\mathcal {X} = \mathsf {var}(T)$ and may write (*T*, *λ*) to emphasize a PT to be projection free. The size |*p*| of a pattern tree is ${\sum }_{t \in V(T)}|\lambda (t)|$.

Well-designed PTs restrict the distribution of variables among their nodes.

### **Definition 1** (Well-Designed Pattern Tree (wdPT))

A PT $(T, \lambda , \mathcal {X})$ is *well-designed* if for every variable *y* ∈*var*(*T*), the set of nodes of *T* where *y* appears is connected.

As an immediate consequence of this restriction, in a wdPT $p = (T, \lambda , \mathcal {X})$, for every variable *y* ∈*var*(*T*) there exists a unique node *t* ∈ *T* such that *y* ∈*var*(*t*) and all nodes $t^{\prime } \in T$ with $y \in \mathsf {var}(t^{\prime })$ are descendants of *t*.

Evaluating a wdPT *p* with free variables $\mathcal {X}$ over a database **D** returns a set *p*(**D**) of mappings $\mu \colon \mathcal {V} \rightarrow \mathsf {dom}(\mathbf {D})$ with $\mathcal {V} \subseteq \mathcal {X}$. We follow the characterization of *p*(**D**) in terms of maximal subtrees introduced by Letelier et al. [17], but borrow the term pp-solution from Kaminski and Kostylev [14].

### **Definition 2** (pp-solution)

For a wdPT *p* = ((*T*, *r*),*λ*) and a database **D**, a mapping $\mu \colon \mathcal {V} \rightarrow \mathsf {dom}(\mathbf {D})$ (with $\mathcal {V} \subseteq \mathsf {var}(T)$) is a *potential partial solution (pp-solution)* to *p* over **D** if there is a subtree $T^{\prime }$ of *T* containing *r* such that $\mu \colon \lambda (T^{\prime }) \rightarrow \mathbf {D}$.

The semantics of wdPTs can now be defined in terms of maximal pp-solutions.

### **Definition 3** (Semantics of wdPTs)

Let $p = (T, \lambda , \mathcal {X})$ be a wdPT, and let $p^{\prime } = (T, \lambda , \mathsf {var}(T))$, i.e., the projection-free wdPT retrieved from *p* by considering all of its variables as free, and let **D** be a database. The set $p^{\prime }(\mathbf {D})$ contains all pp-solutions *μ* to $p^{\prime }$ over **D** such that there exists no pp-solution $\mu ^{\prime }$ to $p^{\prime }$ over **D** that is a proper extension of *μ*.

The set *p*(**D**) is then defined as $p(\mathbf {D}) = \{ \mu |_{\mathcal {X}} \mid \mu \in p^{\prime }(\mathbf {D})\}$.

### *Example 3*

**Fig. 2:**
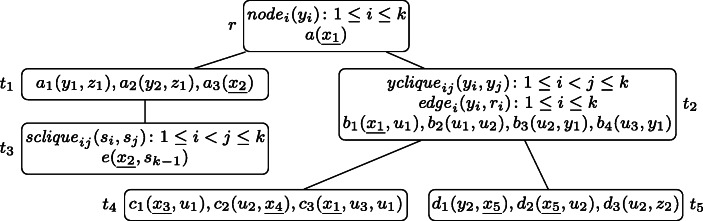
The well-designed pattern tree $p = (T, \lambda , \mathcal {X})$ of Example 3 that will serve as running example through Section 3. The free variables $x_{1}, \dots , x_{5}$ are underlined

Consider the PT $p = (T, \lambda , \mathcal {X})$ depicted in Fig. 2, where *k* may be any integer with *k* ≥ 2, and $\mathcal {X} = \{x_{1}, x_{2}, x_{3}, x_{4}, x_{5}\}$.

All variable occurrences in *p* are connected, thus it is well-designed. If for example the atom *node*_2_(*y*_2_) was missing in the root node, the tree would not be well-designed because of the occurrences of *y*_2_ in both, *t*_1_ and *t*_2_. Similarly, the wdPT in Fig. 1 is also well-designed. If there, in node *t*_3_ one would ask for a review of the airline instead of the seat (i.e., having *λ*(*t*_3_) = *review*(*airline*, *rating*)), the resulting tree would no longer be well-designed, since the variable *airline* does not occur in *t*_2_.

Returning to the wdPT in Fig. 2, consider a database **D** that, for each atom *R*(**v**) in *λ*(*T*) contains one atom $R(1,\dots ,1)$ (i.e., with the value 1 at each position) and in addition the atoms *b*_1_(1,2), *b*_2_(2,2), and *b*_3_(2,1). Then *p*(**D**) = {*μ*_1_, *μ*_2_} where dom$(\mu _{1}) = \{x_{1}, \dots , x_{5}\}$, *dom*(*μ*_2_) = {*x*_1_, *x*_2_}, and *μ*_*i*_(*x*) = 1 for *i* ∈{1,2} and all *x* ∈*dom*(*μ*_*i*_). This is because of the following extensions $\mu _{1}^{\prime }$ and $\mu _{2}^{\prime }$ of *μ*_1_ and *μ*_2_, respectively. For $\mu _{1}^{\prime }$, we have $\mathsf {dom}(\mu _{1}^{\prime }) = \mathsf {var}(T)$ and $\mu _{1}^{\prime }(x) = 1$ for all $x \in \mathsf {dom}(\mu _{1}^{\prime })$, and for $\mu _{2}^{\prime }$ we have $\mathsf {dom}(\mu _{2}^{\prime }) = \mathsf {var}(\lambda (\{r, t_{1}, t_{2}, t_{3}\}))$ with $\mu _{2}^{\prime }(x) = 1$ for all $x \in \mathsf {dom}(\mu _{2}^{\prime })$ except for *u*_1_ and *u*_2_, for which $\mu _{2}^{\prime }(u_{i}) = 2$. Observe that $\mu _{2}^{\prime }$ maps *r*, *t*_1_, *t*_2_, and *t*_3_ into **D**, but cannot be extended to neither *t*_4_ nor *t*_5_.

Orthogonally to wdPTs, *simple* pattern trees restrict the occurrences of relation symbols among their nodes: in a simple pattern tree, no relation symbol is allowed to occur more than once.

### **Definition 4** (Simple PTs)

A PT $p = (T, \lambda , \mathcal {X})$ over *σ* is a *simple pattern tree* if *λ*(*T*) is simple and $\lambda (t) \cap \lambda (t^{\prime }) = \emptyset $ for all $t, t^{\prime } \in T$ with $t \neq t^{\prime }$.

### Parameterized Complexity

We only give a bare-bones introduction to parameterized complexity and refer the reader to [9] for more details. Let Σ be a finite alphabet. A *parameterization* of Σ^∗^ is a polynomial time computable mapping $\kappa \colon {\Sigma }^{*} \rightarrow \mathbb {N}$. A *parameterized problem* over Σ is a pair (*L*, *κ*) where $L \subseteq {\Sigma }^{*}$ and *κ* is a parameterization of Σ^∗^. We refer to *x* ∈Σ^∗^ as the instances of a problem, and to the numbers *κ*(*x*) as the parameters.

A parameterized problem *E* = (*L*, *κ*) belongs to the class *FPT* of *fixed-parameter tractable* problems if there is an algorithm *A* deciding *L*, a polynomial *pol*, and a computable function $f \colon \mathbb {N} \rightarrow \mathbb {N}$ such that the running time of *A* on every input *x* ∈Σ^∗^ is at most *f*(*κ*(*x*)) ⋅*pol*(|*x*|).

In this paper, for classes $\mathcal {P}$ of wdPTs, we study the problem p-Eval($\mathcal {P}$) defined as follows. 
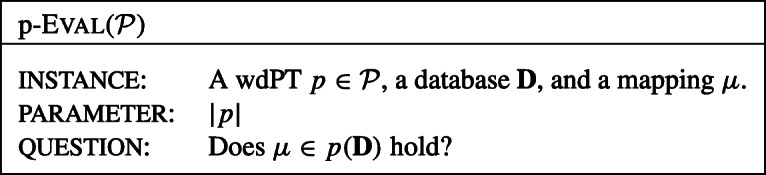


We always assume that the arity of all atoms of the queries in $\mathcal {P}$ is bounded by a constant, i.e., that there is a constant *c* (possibly depending on $\mathcal {P}$) such that no atom in the queries in $\mathcal {P}$ has an arity of more than *c*.

Let *E* = (*L*, *κ*) and $E^{\prime } = (L^{\prime }, \kappa ^{\prime })$ be parameterized problems over Σ and ${\Sigma }^{\prime }$, respectively. An *FPT-reduction* from *E* to $E^{\prime }$ is a mapping $R \colon {\Sigma }^{*} \rightarrow ({\Sigma }^{\prime })^{*}$ such that (1) for all *x* ∈Σ^∗^ we have *x* ∈ *L* if and only if $R(x) \in L^{\prime }$, (2) there is a computable function *f* and a polynomial *pol* such that *R*(*x*) can be computed in time *f*(*κ*(*x*)) ⋅*pol*(|*x*|), and (3) there is a computable function $g \colon \mathbb {N} \rightarrow \mathbb {N}$ such that $\kappa ^{\prime }(R(x)) \leq g(\kappa (x))$ for all *x* ∈Σ^∗^.

Of the rich hardness theory for parameterized problems, we will only use the classes *W*[1] and *coW*[1]. To keep this introduction short, we define a parameterized problem (*L*, *κ*) to be *W*[1]-hard if there is a *W*[1]-hard problem $(L^{\prime }, \kappa ^{\prime })$ that *FPT*-reduces to (*L*, *κ*). We define (*L*, *κ*) to be *coW*[1]-hard if its complement is *W*[1]-hard. It is generally conjectured that *FPT*≠*W*[1] and thus in particular *W*[1]-hard problems and *coW*[1]-hard problems are not in *FPT*. We will take the hardness results for problems $(L^{\prime },\kappa ^{\prime })$ from the literature. One important such problem is the homomorphism problem $\mathrm {p{-}HOM}(\mathcal {C})$ for a class $\mathcal {C}$ of sets of atoms defined as follows 
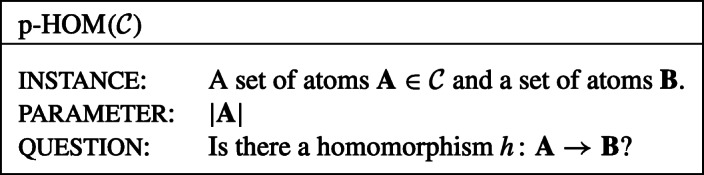


### **Theorem 1** (Grohe [12])

Let $\mathcal {C}$ be a decidable class of sets of atoms. Then $\mathrm {p\textup {-}HOM}(\mathcal {C})$ is in *FPT* if there exists some constant *c* such that the treewidth of the core of each set in $\mathcal {C}$ is bounded by *c*, and *W*[1]-hard otherwise.

Since simple sets of atoms are their own core, this immediately implies that for *simple* sets $\mathcal {C}$ of atoms, $\mathrm {p\textup {-}HOM}(\mathcal {C})$ is in *FPT* if the *treewidth of each set* in $\mathcal {C}$ is bounded by *c*, and *W*[1]-hard otherwise. We remark that this result was in fact shown by Grohe et al. [13] in a predecessor paper of Grohe [12].

## Tractability Conditions for Simple wdPTs

In this section we will introduce our tractability conditions for simple wdPTs. While, as mentioned, these criteria also apply to arbitrary wdPTs, they are not optimal in this case as we will see in Section 6. There we will also show generalizations of the conditions we give here that, for general wdPTs, describe more tractable classes. However, to simplify the presentation, in this section we tailor the definitions towards simple wdPTs.

We start by recalling that in *simple* pattern trees, no relation symbol is allowed to occur more than once. Our overall idea for solving p-Eval($\mathcal {P}$) is as follows: given a wdPT *p*, a database **D**, and a mapping *μ*, construct a set of CQs *q* with free variables **x** and associated databases $\mathbf {D}^{\prime }$ such that *μ* ∈ *p*(**D**) if and only if for at least one of these CQs *q* the tuple *μ*(**x**) is in $q(\mathbf {D}^{\prime })$. We give two tractability criteria ensuring that this algorithm is in *FPT*. Intuitively, one condition guarantees that deciding $\mu (\mathbf {x}) \in q(\mathbf {D}^{\prime })$ is in *PTIME*, while both conditions in combination guarantee that $\mathbf {D}^{\prime }$ can be computed efficiently.

We will state the tractability conditions with respect to a class $\mathcal {P}$ of wdPTs. So in the remainder of this section let $\mathcal {P}$ be an arbitrary but fixed class of wdPTs.

We start with some additional notation and results. First of all, as already observed by Letelier et al. [17], some nodes of a wdPT may not be relevant for the answers returned by the query, in the following sense.

### **Definition 5** (Relevant Nodes)

Let $p = (T, \lambda , \mathcal {X})$ be a wdPT. A node *t* ∈ *T* is *relevant* if there exists a database **D** such that $p(\mathbf {D}) \neq p^{\prime }(\mathbf {D})$ where $p^{\prime }$ is constructed from *p* by removing from *T* the subtree rooted in *t*. We use *relv*(*T*) to denote the set of relevant nodes in *T*.

### *Example 4*

Recall the wdPT from Fig. 2, and consider the node *t*_3_. Given any mapping *μ* on at least *var*(*λ*(*r*) ∪ *λ*(*t*_1_)), whether this mapping can be extended to *t*_3_ or not has no effect on the result, since an extension to *t*_3_ does not include any new free variable. Note, however, that due to the clique of fresh existential variables $s_{1}, \dots , s_{k}$ in *λ*(*t*_3_), deciding whether a mapping can be extended to *t*_3_ is actually expensive. Thus, nodes like *t*_3_ that do not influence any solution can safely be omitted.

Letelier et al. [17] introduced a normal form excluding non-relevant nodes. Here, in order to make our results more explicit, we do not follow this approach but allow wdPTs to contain non-relevant nodes. Luckily, it follows from Letelier et al. [17] that these nodes can be easily detected.

### **Proposition 1** (**Letelier et al.** [17]^1^)

Let $p = (T, \lambda , \mathcal {X})$ be a wdPT. Then a node *t* ∈ *T* is *relevant* if and only if $\mathsf {fvar}(T^{\prime }) \setminus \mathsf {fvar}(\hat t) \neq \emptyset $, where $T^{\prime }$ is the subtree of *T* rooted in *t* and $\hat t$ is the parent node of *t*.

When testing whether a mapping can be extended to a node *t* in a wdPT, the variables shared between *t* and its parent node play a crucial role. First, they describe the relevant domain of the mapping to be extended. Second, the values for these variables are already determined. This not only reduces the number variables in *t* for which a value must be found, but may also allow to partition the atoms in *t* and then test each partition separately instead of all atoms in *t* at once. We call these shared variables the interface of *t*.

### **Definition 6** (Interface $\mathcal {I}(t)$ of a Node)

Let $(T, \lambda , \mathcal {X})$ be a wdPT, *t* ∈ *T* (but not the root node), and $\hat t$ the parent node of *t*. The *interface* $\mathcal {I}(t)$ of *t* is the set $\mathcal {I}(t) = \mathsf {var}(t) \cap \mathsf {var}(\hat t)$. The interface of the root node *r* is $\mathcal {I}(r) = \emptyset $.

It was already remarked, e.g., by Barceló et al. [3] and Kröll et al. [16] that restrictions on the number of variables shared between different sets of nodes can be used to define tractable classes. The above definition however differs slightly from the notion of interfaces in these works. E.g., in Kröll et al. [16], the interface of a node describes the set of variables shared between the node and any of its neighbors, while here it is restricted to the variables shared with its parent node.

Restrictions on the size of node interfaces turn out to be quite coarse, and we provide more fine grained tractability criteria here. To this end, we implement the idea of partitioning the set of atoms in a node using its interface. For this, we recall the notion of *S*-components from Durand and Mengel [8]. Originally, they were defined for graphs and then extended to sets of atoms. Since we will only use *S*-components of sets of atoms, we provide their definition directly, omitting the graph case. Let **A** be a set of atoms and $S \subseteq \mathsf {var}(\mathbf {A})$ a set of variables. Consider the dual graph *G*_**A**_ = (*V*_**A**_, *E*_**A**_) with $V_{\mathbf {A}} = \{\tau \in \mathbf {A} \mid \mathsf {var}(\tau ) \nsubseteq S\}$ and *E*_**A**_ = {{*τ*_*i*_, *τ*_*j*_}∣*τ*_*i*_, *τ*_*j*_ ∈**A** s.t. (*var*(*τ*_*i*_) ∩*var*(*τ*_*j*_)) ∖ *S*≠*∅*}. The connected components of *G*_**A**_ are the *S*-components of **A**.

### **Definition 7** (Kroll et al.¨ [16]; Node Components)

Let $p = (T, \lambda , \mathcal {X})$ be a wdPT and *t* ∈ *T*. The set of *node components* $\mathcal {N}\mathcal {C}({t})$ *oft* is a set of sets of atoms, defined as the union of:

1. The set $\{ \{\tau \} \mid \tau \in \lambda (t) \text { and } \mathsf {var}(\tau ) \subseteq \mathcal {I}(t) \}$ consisting of singleton sets for every atom *τ* ∈ *λ*(*t*) which contains only “interface variables”, i.e., variables from $\mathcal {I}(t)$.
2. The set of all $\mathcal {I}(t)$-components of *λ*(*t*).

In the following, node components of *type (1)* are the singleton sets of condition 1 and node components of *type (2)* are the $\mathcal {I}(t)$-components of condition 2.

### *Example 5*

**Fig. 3:**
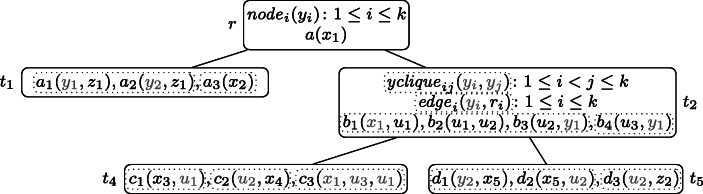
The well-designed pattern tree from Fig. 2, with the non-relevant node *t*_3_ omitted and the interface variables at each node grayed to emphasize the node components, which are depicted by the dotted boxes (see Example 5)

Fig. 3 shows again the wdPT *p* from Example 3, but omitting the non-relevant node *t*_3_. In addition, at each node the node components are depicted by dotted boxes. To emphasize the difference between interface- and non interface variables and to highlight which variables connect atoms to node components, the interface variables at each node are grayed.

Consider the node *t*_2_, which has the following node components: each atom *yclique*_*ij*_(*y*_*i*_, *y*_*j*_) forms a node component of type (1) since all variables *y*_*i*_ occur also in *r*. In addition, there are several node components of type (2): each atom *edge*_*i*_(*y*_*i*_, *r*_*i*_) is such a node component (they contain a variable in $\mathsf {var}(t_{2}) \setminus \mathcal {I}(t_{2})$ but do not share such a variable with another atom), and there are the two node components {*b*_1_(*x*_1_, *u*_1_),*b*_2_(*u*_1_, *u*_2_),*b*_3_(*u*_2_, *y*_1_)} and {*b*_4_(*u*_3_, *y*_1_)}. Observe that these two sets are connected by *y*_1_, but $y_{1} \in \mathcal {I}(t_{2})$ separates them into two node components. In contrast, the atoms *b*_1_(*x*_1_, *u*_1_), *b*_2_(*u*_1_, *u*_2_), and *b*_3_(*u*_2_, *y*_1_) are connected by *u*_1_ and *u*_2_, which are not in $\mathcal {I}(t_{2})$.

Also, note the effect of considering interface variables and node components instead of looking at the complete node at once. For example, the Gaifman graph of *λ*(*t*_2_) has a treewidth of *k*. Thus, finding, e.g., values for $r_{1}, \dots , r_{k}$ is a hard problem. However, by taking into account interface variables and node components, each of the resulting sets has a treewidth of 1. Therefore, the existence of an extension of some mapping on the interface variables to each node component independently can be decided efficiently.

To understand why node components are essential for our results, recall that solutions to wdPTs must be maximal, i.e., they map some subtree into the database, but cannot be extended to map some child node of this subtree into the database as well. But such an extension to a node exists if and only if the mapping can be extended to all of its node components. Thus, instead of testing extensions to the complete node at once (which might be intractable), we test the maximality of a mapping independently for each node component (which might be tractable). This is possible because for all variables shared between any two node components, the values are already determined by the mapping to be extended. Extensions to different node components are thus independent of each other.

For node components, we are in particular interested in the contained interface variables.

### **Definition 8** (Interface of a Node Component)

For a wdPT $(T, \lambda , \mathcal {X})$ and a node *t* ∈ *T*, the *interface of a node component* $\mathbf {S} \in \mathcal {N}\mathcal {C}({t}) $ is $\mathcal {I}({\mathbf {S}}, t) = (\mathcal {I}(t) \cap \mathsf {var}(\mathbf {S}))$, and the *existential* interface is $\mathcal {I}^{\exists }(\mathbf {S}, t) = \mathcal {I}(\mathbf {S}, t) \setminus \mathcal {X}$.

We are now ready to formulate our first tractability condition. Intuitively, it guarantees that for each node component **S**, given some mapping on the variables in its interface, one can decide in polynomial time whether this mapping can be extended to map all atoms in the node component into a given database. It exploits (and formalizes) the fact that once a mapping on $\mathcal {I}(\mathbf {S}, t)$ is given, these variables can be treated as constants. 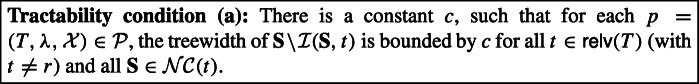


### *Example 6*

To demonstrate the situation after introducing condition (a), let *p*_*k*_ be the wdPT *p* from Example 5 parameterized by *k*, let $\mathcal {P} = \{p_{k} \mid k \geq 2\}$, and let $T^{\prime } = T[\{r,t_{1},t_{2}\}]$. Assume, for some *k* ≥ 2, in order to test if some mapping is a solution to *p*_*k*_, we would like to verify whether some mapping $\mu ^{\prime }$ with $\mathsf {dom}(\mu ^{\prime }) = \mathsf {var}(T^{\prime })$ is a maximal pp-solution. Deciding whether it is a pp-solution is easy, and because $\mathcal {P}$ satisfies tractability condition (a), testing if there exists in both, *t*_4_ and *t*_5_, a node component to which $\mu ^{\prime }$ cannot be extended is feasible in polynomial time as well. In fact, $\mathcal {N}\mathcal {C}(t_{4}) = \{\{c_{1}(x_{3},u_{1})\},$ {*c*_2_(*u*_2_, *x*_4_)},{*c*_3_(*x*_1_, *u*_3_, *u*_1_)}} and $\mathcal {N}\mathcal {C}({t_{5}}) = \{\{d_{1}(y_{2}, x_{5}),$ *d*_2_(*x*_5_, *u*_2_)}, {*d*_3_(*u*_2_, *z*_2_)}} (this holds for every $p_{k} \in \mathcal {P}$). However, there may exist an exponential number of pp-solutions on $T^{\prime }$ ($T^{\prime }$ contains 2*k* + 4 existential variables). Thus, testing one mapping on $\mathsf {var}(T^{\prime })$ after the other is not feasible.

One way to overcome the problem sketched in the example is to not have two separate tests for being a pp-solution and being maximal. To combine these tests, we first compute for each node component the set of mappings on its interface variables that do not extend to the component, and then require pp-solutions to be consistent with these mappings.

It turns out that this idea can be encoded as an evaluation problem for CQs. One important step in this encoding is to introduce new relations, one for each node component, that store those mappings on the interface variables that cannot be extended to the component. In order for the resulting CQ evaluation problem to be in polynomial time, we require two properties. First, the resulting CQs must be from some tractable fragment of CQ evaluation, and, second, the size of the newly added relations must be at most polynomial. One way to achieve the second goal is to restrict the arity of these relations. Towards the first goal, since the bodies of the resulting CQs are simple sets of atoms, tractability for the evaluation problem holds if these queries have bounded treewidth. As it turns out, a bound on the treewidth also implies a bound on the arity of the new relations, and thus represents our second tractability condition.

To formalize the construction, we introduce the notion of a *component interface atom*. For a wdPT $(T, \lambda , \mathcal {X})$, a subtree $T^{\prime }$ of *T*, a node $t \in ch(T^{\prime })$, and node component $\mathbf {S} \in \mathcal {N}\mathcal {C}(t)$, let the interface atom be an atom *R*(**v**) where **v** contains the variables in $\mathcal {I}^{\exists }({\mathbf {S}}, t)$ and *R* is a fresh relation symbol. For a node component **S**, we use *cia*(**S**) to refer to the corresponding interface atom *R*(**v**). Observe that these definitions imply *cia*(**S**) = *R*() in case $\mathcal {I}^{\exists }(\mathbf {S}, t) = \emptyset $.

The intuition for *cia*(**S**) is that for each node component, we get one atom that covers exactly the variables in $\mathcal {I}^{\exists }({\mathbf {S}}, t)$. The free variables in the interface can be excluded from the considerations since a fixed value is provided for them as part of the input.

### *Example 7*

Recall again the wdPT depicted in Fig. 3 and consider the node *t*_1_. It contains two node components: **S**_1_ = {*a*_1_(*y*_1_, *z*_1_), *a*_2_(*y*_2_, *z*_1_)} and **S**_2_ = {*a*_3_(*x*_2_)}. Observe that whether **S**_2_ can be mapped into some database **D** is completely independent of the interface variables. Thus, $\mathsf {cia}(\mathbf {S}_{2}) = R_{\mathbf {S}_{2}}()$. However, for **S**_1_ the values of *y*_1_ and *y*_2_ influence whether **S**_1_ may be mapped. Thus, we get $\mathsf {cia}({\mathbf {S}_{1}}) = R_{\mathbf {S}_{1}}(y_{1}, y_{2})$. In case of the node component {*c*_3_(*x*_1_, *u*_3_, *u*_1_)} of *t*_4_, observe that we can assume some fixed value *μ*(*x*_1_) for *x*_1_, and thus can reduce the atom *c*_3_(*μ*(*x*_1_),*u*_3_, *u*_1_) according to this value. We therefore get as interface atom $R_{c_{3}}(u_{3}, u_{1})$, with the corresponding database being computed based on $c_{3}^{x_{1}=\mu (x_{1})}(u_{3}, u_{1})$.

Since we are looking for *one* pp-solution that cannot be extended to *any* child node, combining the two tests as sketched means that we must test all children simultaneously instead of individually. However, since each CQ tests only one node component for each child, we need one CQ for each possible combination, leading to our second tractability condition. 
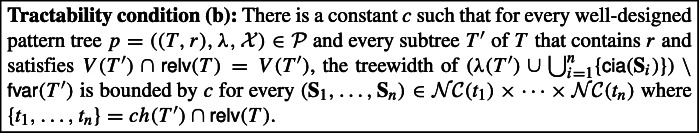


The following example breaks down condition (b) to demonstrate its intuition.

### *Example 8*

Recall the setting in Example 6, as well as the database instance **D** from Example 3. Let *μ* be a mapping with *dom*(*μ*) = {*x*_1_, *x*_2_}, and *μ*(*x*_1_) = *μ*(*x*_2_) = 1. Assume that, in order to show that *μ* ∈ *p*_*k*_(**D**) for any *k* ≥ 2 we are looking for a pp-solution for $T^{\prime }$ that does not extend neither to the node component **S**_1_ = {*c*_1_(*x*_3_, *u*_1_)} of *t*_4_ (and thus not to *λ*(*t*_4_)), nor to the node component **S**_2_ = {*d*_1_(*y*_2_, *x*_5_),*d*_2_(*x*_5_, *u*_2_)} of *t*_5_ (and thus not to *λ*(*t*_5_)). The idea is to construct a CQ $\textit {Ans}(x_{1},x_{2}) \leftarrow \lambda (T^{\prime }) \cup \{R_{1}(u_{1}), R_{2}(y_{2},u_{2})\}$, where *R*_1_(*u*_1_) and *R*_2_(*y*_2_, *u*_2_) are the component interface atoms for **S**_1_ and **S**_2_, respectively. Observe that this query has exactly the structure described in condition (b), illustrating the motivation for the definition of this condition. Also, note that because of the *yclique*_*ij*_-atoms, $\mathcal {P}$ does not satisfy tractability condition (b).

The query is evaluated over the instance **D** extended by relations for *R*_1_ and *R*_2_. As mentioned, these relations contain all values that cannot be extended to map **S**_1_ and **S**_2_ into **D**, respectively. For **S**_1_, we get {*R*_1_(2)} (since *c*_1_(1,1) ∈**D**, thus a mapping assigning 1 to *u*_1_ could be extended to map **S**_1_ into **D**), and for **S**_2_ we get {*R*_2_(1,2),*R*_2_(2,1),*R*_2_(2,2)}.

Consider the mapping $\mu ^{\prime }$ with $\mathsf {dom}(\mu ^{\prime }) = \mathsf {var}(\lambda (\{r, t_{1}, t_{2}, t_{3}\}))$ and $\mu ^{\prime }(x) = 1$ for all $x \in \mathsf {dom}(\mu ^{\prime })$ except for *u*_1_ and *u*_2_, for which $\mu ^{\prime }(u_{i}) = 2$. Now the mapping $\mu ^{\prime }$ witnesses the fact that (1,1) is an answer to the query over **D**, and thus $\mu _{2}^{\prime }$ is a maximal pp-solution also witnessing *μ* ∈ *p*_*k*_(**D**).

The main result of this paper is that the conditions (a) and (b) characterize exactly the classes of simple wdPTs which can be evaluated efficiently.

### **Theorem 2**

Assume that *FPT*≠*W*[1], and let $\mathcal {P}$ be a decidable class of simple wdPTs of bounded arity. Then the following statements are equivalent.

1. The tractability conditions (a) and (b) hold for $\mathcal {P}$.
2. p-Eval($\mathcal {P}$) is in *FPT*.

We will show the upper bound of Theorem 2 in Section 4, where we describe how the different ideas described so far can be combined to an *FPT* algorithm, while the lower bounds will be shown in Section 5.

But before we turn to the proof of Theorem 2, let us interpret the result in the setting without projections to better understand the influence of projection. First note that in that case, by Definition 8, we have $\mathcal {I}^{\exists }({\mathbf {S}}, t) =\emptyset $ for every *t* ∈ *T* and every $\mathbf {S}\in \mathcal {N}\mathcal {C}({t}) $. Thus, all atoms *cia*(**S**) (for any node component **S**) are of arity 0 as are all atoms in $(\lambda (T^{\prime }) \cup \bigcup _{i=1}^{n} \{\mathsf {cia}({\mathbf {S}_{i}}) \}) \setminus \mathsf {fvar}(T^{\prime })$. Tractability condition (b) is therefore void in this setting, leaving only (a) as a useful condition in the projection free case. This immediately implies the following corollary.

### **Corollary 1**

Assume that *FPT*≠*W*[1], and let $\mathcal {P}$ be a decidable class of simple wdPTs of bounded arity without projections. Then p-Eval($\mathcal {P}$) is in *FPT* if and only if tractability condition (a) holds for $\mathcal {P}$.

We remark that Corollary 1 could also be inferred as a special case of the main result of Romero [24]. Stating the corollary explicitly here lets us better understand the role of projection for our problem: in fact, the role of condition (a) is essentially to deal with the complexity that we already have without projection, while condition (b) is necessary to deal with the additional source of hardness that is introduced by projections and does not appear without them.

Since it will simplify the discussion in the upcoming sections, we conclude the section by explicitly working out a third tractability condition already mentioned above in the discussion towards tractability condition (b). As described there, at some point we extend a given database by relations for the atoms *cia*(**S**) that contain for the corresponding node component all mappings on its existential interface that cannot be extended to a mapping on the whole node component. To guarantee that all these relations are of polynomial size, we restrict the number of variables in the existential interfaces of the node components by some constant *c*. We formalize this notion in terms of a suitable width measure.

### **Definition 9** (Component Width)

Let $p = (T, \lambda , \mathcal {X})$ be a wdPT, *t* ∈ *T*, and $\mathbf {S} \in \mathcal {N}\mathcal {C}({t}) $. The *width* of the node component **S** is $|\mathcal {I}^{\exists }({\mathbf {S}}, t) |$. For a node *t* ∈ *T*, the *component width* of *t* is the maximum width over all node components **S** of *t*. The component width of *p* is the maximum component width over all *t* ∈*relv*(*T*).

By the definition of the treewidth of a set of atoms – specifically by the fact that in the Gaifman graph all variables occurring together in an atom form a clique – and the fact that for the existential interface of each node component its variables occur together in some *cia*(**S**)-atom, the number of variables in any existential interface is bound by the treewidth of the CQs defined in condition (b). Thus, we get the following corollary.

### **Corollary 2**

Let $\mathcal {P}$ be a class of wdPTs that satisfies tractability condition (b) for some constant *c*. Then, for every $p \in \mathcal {P}$, the component width of *p* is at most *c* + 1.

## The FPT Algorithm

Having defined the tractability conditions, we now show how they are used in the *FPT*-algorithm for p-Eval($\mathcal {P}$) outlined in Algorithm 1. 
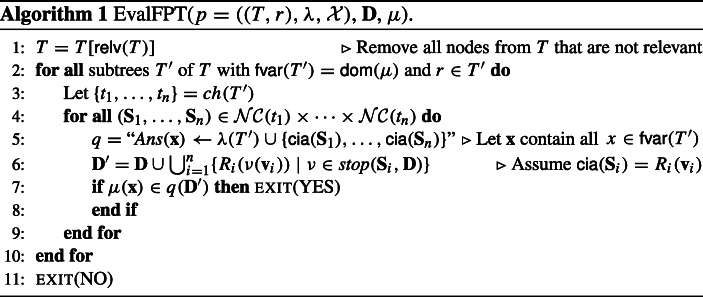


The missing ingredient of Algorithm 1 that we have not yet introduced is *stop*(**S**, **D**) for a node component **S** and a database **D** which we explain now. Recall that we said earlier that the intention of the node components is to ensure a mapping to be maximal not by testing for extensions to the complete node, but to do these tests for smaller, independent units.

The idea of how to realize this is to store in $\mathbf {D}^{\prime }$ for each node component **S** those variable assignments *ν* to the variables in its existential interface such that there exists no extension homomorphism $\nu ^{\prime } \colon \mathbf {S} \rightarrow \mathbf {D}$ of *ν* ∪ *μ*. These are the values stored in *stop*(**S**, **D**).

In more detail, for a wdPT $((T,r), \lambda , \mathcal {X})$, a subtree $T^{\prime }$ of *T* containing *r*, a child node $t \in ch(T^{\prime })$, node component $\mathbf {S} \in \mathcal {N}\mathcal {C}({t})$, a database **D**, and a mapping $\mu \colon \mathsf {fvar}(T^{\prime }) \rightarrow \mathsf {dom}(\mathbf {D})$, consider the set $\textit {extend}(\mathbf {S}, \mathbf {D}) = \{ \eta \colon \mathcal {I}^{\exists }({\mathbf {S}}, t) \rightarrow \mathsf {dom}(\mathbf {D}) \mid \text {there exists a homomorphism } \eta ^{\prime }\colon \mathbf {S} \rightarrow \mathbf {D} \text { extending } \eta \text { and } \mu |_{\mathsf {var}(\mathbf {S})} \}$. So *extend* contains exactly those mappings on $\mathcal {I}^{\exists }(\mathbf {S}, t)$ that *can* be extended in a way that is compatible with *μ* and maps **S** into **D**. We thus set $\textit {stop}(\mathbf {S}, \mathbf {D}) = \{ \nu \colon \mathcal {I}^{\exists }(\mathbf {S}, t) \rightarrow \mathsf {dom}(\mathbf {D}) \mid \nu \notin \textit {extend}(\mathbf {S}, \mathbf {D})\}$.

With this in place, we describe the idea of Algorithm 1. Recall that, given *μ*, we have to find a mapping $\mu ^{\prime }$ extending *μ* that is (1) a pp-solution, and (2) maximal. Because of the existential variables, there may be exponentially many subtrees $T^{\prime }$ of *T* containing *r* with $\mathsf {fvar}(T^{\prime }) = \mathsf {dom}(\mu )$, each being a potential candidate for witnessing (1) and (2). After removing all irrelevant nodes in line 1 (they might make evaluation unnecessarily hard, cf. Example 4), we thus check each of these subtrees (line 2).

If the required mapping $\mu ^{\prime }$ exists, then, as discussed earlier, for each child node of $T^{\prime }$ there exists at least one node component to which $\mu ^{\prime }$ cannot be extended. Not knowing which node components these are, the algorithm iterates over all possible combinations (line 4). In lines 5–7, the algorithm now checks whether there exists an extension of *μ* that maps all of $\lambda (T^{\prime })$ into **D** (ensured by adding $\lambda (T^{\prime })$ to *q*), but none of the node components $\mathbf {S}_{1}, \dots , \mathbf {S}_{n}$. The latter property is equivalent to asking that $\mu ^{\prime }$ must assign a value to the existential interface variables of each **S**_*i*_ that cannot be extended. This is guaranteed by adding the atoms *cia*(**S**_*i*_) to *q* and providing in $\mathbf {D}^{\prime }$ exactly the values from *stop*(**S**_*i*_, **D**). Observe that the CQ *q* in line 5 contains all $x \in \mathsf {fvar}(T^{\prime })$, and that replacing them by *μ*(*x*) is only part of the evaluation strategy in line 7.

Most of the central components and ideas of the algorithm have already been demonstrated in isolation in the examples throughout Section 3. The next example brings together all these ideas by illustrating how the algorithm works.

### *Example 9*

Consider again the wdPT $p = (T, \lambda , \{x_{1}, \dots , x_{5}\})$ depicted in Fig. 2, but without the atoms *yclique*_*ij*_(*y*_*i*_, *y*_*j*_) for 1 ≤ *i* < *j* ≤ *k*. The resulting tree now satisfies conditions (a) and (b). Assume we want to decide *μ* ∈ *p*(**D**) for the mapping *μ* with *μ*(*x*_1_) = 1 and *μ*(*x*_2_) = 2 and **D** = $\{R(1, \dots , 1) \mid R(\mathbf {v}) \in (\lambda (T) \setminus \{a_{3}(x_{2})\})\} \cup \{ a_{3}(2)\} \cup \text {}$ {*b*_1_(1,3),*b*_2_(3,3),*b*_3_(3,1),}∪{*b*_1_(2,2),*b*_2_(2,2),*b*_3_(2,2)}∪ {*c*_1_(2,2),*c*_1_(3,3)}∪{*c*_2_(3,3)}∪ {*d*_1_(2,1),*d*_1_(2,2),*d*_1_(3,1),*d*_2_(1,3),*d*_2_(2,2)}.

In a first step, the algorithm now reduces *T* by removing the non-relevant node *t*_3_. Thus, from now on *T* corresponds to the wdPT shown in Fig. 3 (again, without the *yclique*_*ij*_(*y*_*i*_, *y*_*j*_) atoms in *t*_2_).

Next, there exist two subtrees $T^{\prime }$of *T* with $\mathsf {fvar}(T^{\prime }) = \mathsf {dom}(\mu ) = \{x_{1}, x_{2}\}$. These are the subtrees *T*_1_ = *T*[{*r*, *t*_1_}] and *T*_2_[{*r*, *t*_1_, *t*_2_}], respectively. Starting with *T*_1_, we get *ch*(*T*_1_) = {*t*_2_}. Without the *yclique*_*ij*_-atoms, the node *t*_2_ has *k* + 2 node components. Since *t*_2_ is the only child of *T*_1_, the algorithm simply iterates over these *k* + 2 node components (line 4). We distinguish four different kinds of node components in *t*_2_.

For components **S** of the form *edge*_*i*_(*y*_*i*_, *r*_*i*_), the component interface atoms are $\mathsf {cia}({\mathbf {S}}) = R^{\textit {edge}}_{i}(y_{i})$. Thus, the CQ *q* is $\textit {Ans}(x_{1}, x_{2}) \leftarrow \lambda (T_{1}) \cup \{R^{\textit {edge}}_{i}(y_{i})\}$. Also, *extend*(**S**, **D**) = {*ν*} with *ν*(*y*_*i*_) = 1, and thus $\mathbf {D}^{\prime } = \mathbf {D} \cup \{R^{\textit {edge}}_{i}(2),$ $R^{\textit {edge}}_{i}(3)\}$. Clearly, we get $(1,2) \notin q(\mathbf {D}^{\prime })$.

For the node component **S** = {*b*_1_(*x*_1_, *u*_1_),*b*_2_(*u*_1_, *u*_2_),*b*_3_(*u*_2_, *y*_1_)}, we get *cia*(**S**) = *R*_1_(*y*_1_) and therefore *q* is $\textit {Ans}(x_{1}, x_{2}) \leftarrow \lambda (T_{1}) \cup \{R_{1}(y_{1})\}$, with $\mathbf {D}^{\prime } = \mathbf {D} \cup \{R_{1}(2), R_{1}(3)\}$. The reason *R*_1_(2) is in this list is that *μ*(*x*_1_) = 1 is already given. Observe that because of $\{b_{1}(2,2), b_{2}(2,2), b_{3}(2,2)\} \subseteq \mathbf {D}$, in principle a mapping *ν*(*y*_1_) = 2 can be extended to a mapping on **S**. However, because of *μ*(*x*_1_) = 1, such a mapping would not be compatible with *μ*, since mapping *b*_1_(*x*_1_, *u*_1_) to *b*_1_(2,2) is not an option. As a result, again $(1,2) \notin q(\mathbf {D}^{\prime })$.

We omit the similar case for the last node component **S** = {*d*_4_(*u*_3_, *y*_1_)}.

Hence, *T*_1_ does not witness *μ* being a solution, and therefore *T*_2_ is tested next (line 2). The children of *T*_2_ are *t*_4_ and *t*_5_. As already laid out in Example 6, $\mathcal {N}\mathcal {C}(t_{4}) = \{\{c_{1}(x_{3},u_{1})\}, \{c_{2}(u_{2},x_{4})\}, \{c_{3}(x_{1},u_{3},u_{1})\}\}$ and $\mathcal {N}\mathcal {C}({t_{5}}) = \{\{d_{1}(y_{2}, x_{5}),$*d*_2_(*x*_5_, *u*_2_)}, {*d*_3_(*u*_2_, *z*_2_)}}. This gives six different combinations of node components from *t*_4_ and *t*_5_. For the purpose of illustration, we give the component interface atoms and database extensions for each component.

For **S**_1_ = {*c*_1_(*x*_3_, *u*_1_)}, we get *cia*(**S**_1_) = *R*_1_(*u*_1_) and **D**_1_ = {}; for **S**_2_ = {*c*_2_(*u*_2_, *x*_4_)}, we get *cia*(**S**_2_) = *R*_2_(*u*_2_) and **D**_2_ = {*R*_2_(2)}; and for **S**_3_ = {*c*_3_(*x*_1_, *u*_3_, *u*_1_)} we get *cia*(**S**_3_) = *R*_3_(*u*_3_, *u*_1_) with $\mathbf {D}_{3} = \{R(a,b) \mid (a,b) \in (\{1,2,3\}^{2} \setminus (1,1))\}$. For the node components of *t*_5_ we get for **S**_4_ = {*d*_1_(*y*_2_, *x*_5_), *d*_2_(*x*_5_, *u*_2_)} the atom *cia*(**S**_4_) = *R*_4_(*y*_2_, *u*_2_) and **D**_4_ = {*R*_4_(1,2), *R*_4_(3,2)}. Finally, for **S**_5_ = {*d*_3_(*u*_2_, *z*_2_)}, we get *cia*(**S**_5_) = *R*_5_(*u*_2_) and **D**_5_ = {*R*_5_(2), *R*_5_(3)}.

Thus, for the first combination (**S**_1_, **S**_4_) we get *q* as $\textit {Ans}(x_{1}, x_{2}) \leftarrow \lambda (T_{2}) \cup \{R_{1}(u_{1}), R_{4}(y_{2}, u_{2})\}$ and $\mathbf {D}^{\prime } = \mathbf {D} \cup \mathbf {D}_{1} \cup \mathbf {D}_{4}$. Because of **D**_1_ = *∅*, clearly $q(\mathbf {D}^{\prime }) = \emptyset $, and thus $(1,2) \notin q(\mathbf {D}^{\prime })$. By the same argument, this is also true for the combination (**S**_1_, **S**_5_).

For the combination (**S**_2_, **S**_4_), we get *q* as $\textit {Ans}(x_{1}, x_{2}) \leftarrow \lambda (T_{2}) \cup \{R_{2}(u_{2}),$ *R*_4_(*y*_2_, *u*_2_)} and $\mathbf {D}^{\prime } = \mathbf {D} \cup \mathbf {D}_{2} \cup \mathbf {D}_{4}$. Observe that **D**_2_ and **D**_4_ do not contain a compatible value for *u*_2_. Thus, $q(\mathbf {D}^{\prime })$ is again empty, showcasing that there must be a combined check for the node components of different nodes, and that this cannot be done in isolation.

For (**S**_2_, **S**_5_), now **D**_2_ and **D**_5_ contain a compatible value for *u*_2_, namely 2. However, by mapping *u*_2_ to 2, there is no way to map *t*_2_ into **D** such that *x*_1_ is mapped to 1. Thus, also in this case $(1,2) \notin q(\mathbf {D}^{\prime })$.

For (**S**_3_, **S**_4_), again $(1,2) \notin q(\mathbf {D}^{\prime })$: mapping all existential variables to 1 clearly does not allow to map neither *R*_3_(*u*_3_, *u*_1_) nor *R*_4_(*y*_2_, *u*_2_) into $\mathbf {D}^{\prime }$. The only other way to map *λ*(*t*_2_) into $\mathbf {D}^{\prime }$ is by mapping *y*_1_ to 1 and *u*_2_ to 3. However, such a mapping cannot map *R*_4_(*y*_2_, *u*_2_) into $\mathbf {D}^{\prime }$.

Finally, for (**S**_3_, **S**_5_), it can now be checked that $(1,2) \in q(\mathbf {D}^{\prime })$, witnessed by the mapping *μ* with *μ*(*x*_1_) = 1, *μ*(*x*_2_) = 2, *μ*(*u*_1_) = *μ*(*u*_2_) = 3, and *μ*(*v*) = 1 for all remaining variables *v* ∈*var*(*T*_2_). Thus, in line 7 the algorithm returns YES.

In order to see that this indeed gives an *FPT* algorithm in case tractability conditions (a) and (b) are satisfied, note that condition (b) ensures that the arity of each of the new relations for the atoms *cia*(**S**) is at most *c* + 1 (cf. Corollary 2). Thus, the size of these relations (and thus the number of possible mappings in *stop*(**S**, **D**)) is at most |*dom*(**D**)|^(*c*+ 1)^. Next, condition (a) ensures that for each mapping $\nu \colon \mathcal {I}^{\exists }({\mathbf {S}}, t) \rightarrow \mathsf {dom}(\mathbf {D})$ deciding membership in *stop*(**S**, **D**) is in *PTIME*. Observe that the variables in $\mathcal {I}({\mathbf {S}}, t) $ are not considered in the computation of the treewidth since a fixed value is provided for them, thus they can be treated as constants. Finally, condition (b) also ensures that the test in line 7 is feasible in polynomial time. Again, since a fixed value is provided for the domain of *μ*, these variables can be treated as constants.

We note that the algorithm is an extension and refinement of the *FPT* algorithm of Kröll et al. [16]. An inspection of that paper reveals that the conditions provided there imply our tractability conditions (a) and (b), but there is no implication in the other direction. In fact, our conditions explicitly describe the crucial properties of their restrictions that ensure the problem is in *FPT*. From Algorithm 1 we thus derive the following result.

### **Lemma 1**

Let $\mathcal {P}$ be a decidable class of wdPTs. If the tractability conditions (a) and (b) hold for $\mathcal {P}$, then p-Eval($\mathcal {P}$) can be solved in *FPT*.

The correctness of the algorithm follows immediately from the previous discussion. For the runtime, in addition to what was already discussed, the number of loop-iterations in lines 2 and 4 is bounded by a function in the size of *p*, which is the parameter for the problem.

## Optimality of the Tractability Conditions

We now show that both tractability criteria are necessary, and thus finish the proof of Theorem 2. We provide individual results for both conditions. In addition, we show that the bound on the component width is necessary (and not just a side effect), which will turn out to be a useful result for proving that tractability condition (b) is necessary.

### **Lemma 2**

Let $\mathcal {P}$ be a decidable class of simple wdPTs of bounded arity such that tractability condition (a) is not satisfied. Then p-Eval($\mathcal {P}$) is *coW*[1]-hard.

### *Proof*

For a wdPT $p \in \mathcal {P}$, let the *relevant components set* *rcs*(*p*) contain all the sets $\mathbf {S} \setminus \mathcal {I}({\mathbf {S}}, t) $ as defined in tractability condition (a). Moreover, let $\mathsf {rcs}(\mathcal {P}) = \bigcup _{p \in \mathcal {P}}\mathsf {rcs}(p)$. We will—by an *FPT*-reduction—reduce $\mathrm {p\textup {-}HOM}(\mathsf {rcs}(\mathcal {P}))$ to the complement of p-Eval($\mathcal {P}$) . The result then follows from Theorem 1, as $\mathsf {rcs}(\mathcal {P})$ does not have bounded treewidth by assumption.

Consider an instance **E**, **F** of $\mathrm {p\textup {-}HOM}(\mathsf {rcs}(\mathcal {P}))$. As a first step, find $p = ((T,r), \lambda , \mathcal {X}) \in \mathcal {P}$, a node *t* ∈*relv*(*T*) such that *t*≠*r*, and a node component $\mathbf {S} \in \mathcal {N}\mathcal {C}(t)$ such that $\mathbf {E} = \mathbf {S} \setminus \mathcal {I}(\mathbf {S}, t)$. They exist by assumption and, since $\mathcal {P}$ is decidable, can be computed as follows: enumerate all possible candidate triples *p*, *t* and **S**, check if $p\in \mathcal {P}$ and if so verify the conditions on *p*, *t* and **S**. Since *p* exists by assumption and all other steps are computable, this procedure eventually yields the desired triple.

Since *t* is relevant, either for $t^{\prime } = t$ or some descendant $t^{\prime }$ of *t* we have $\mathsf {fvar}(t^{\prime }) \setminus \mathsf {fvar}(\mathsf {branch}(t^{\prime })) \neq \emptyset $. Among all possible candidates, pick some $t^{\prime }$ at a minimal distance to *t*.

We next define a database **D** over the set of relation symbols in *p*. For the description of **D**, for all relation symbols *R* occurring in any atom *R*(**v**) ∈ *λ*(*T*), we will assume that **v** contains only variables, i.e., elements from ${\mathcal Var}$. We implicitly assume that for all positions where **v** contains a constant, all atoms in *R*^**D**^ contain the same constant as in **v**. Recall that we deal with simple wdPTs, thus each relation symbol occurs at most once within *λ*(*T*). Also recall that, for any node $\bar t \in T$, $\mathsf {branch}(\bar t)$ contains the nodes on the path from *r* to the parent node of $\bar t$, while $\mathsf {cbranch}(\bar t)$ in addition includes $\bar t$ itself. In the following, let $d \in {\mathcal Const}$ be some fresh value not occurring in *dom*(**F**).

For each relation symbol *R* mentioned outside of $\lambda (\mathsf {cbranch}(t^{\prime }))$, let *R*^**D**^ = *∅*. For each relation symbol *R* mentioned in *λ*(*branch*(*t*)), let $R^{\mathbf {D}} = \{ R(d, \dots , d)\}$. For each relation symbol *R* mentioned in $\lambda (\mathsf {cbranch}(t^{\prime }) \setminus \mathsf {branch}(t)) \setminus \mathbf {S}$, let *k* be the arity of *R* and *R*^**D**^ = {*R*(**v**)∣**v** ∈ (*dom*(**F**) ∪{*d*})^*k*^}.

For each relation symbol *R* mentioned in **S**, observe that there exists a relation symbol $R^{\prime }$ in **E** that was derived from *R* when computing $\mathbf {S} \setminus \mathcal {I}(\mathbf {S}, t)$. The idea is now to use *R*^**D**^ to simulate the atoms with relation symbol $R^{\prime }$ in **F** by padding the additional fields with *d*. Thus, let *k* be the arity of *R*, let *m* be the arity of $R^{\prime }$, let $\{i_{1}, \dots , i_{\ell }\} \subseteq \{1, \dots , k\}$ be those positions of *R* containing values from $\mathcal {I}({\mathbf {S}}, t) $, and $\{o_{1}, \dots , o_{m}\} = \{1, \dots , k\} \setminus \{i_{1}, \dots , i_{\ell }\}$ those positions of *R* that contain values from $\mathsf {var}(\mathbf {S}) \setminus \mathcal {I}({\mathbf {S}}, t)$. Then, for every $R^{\prime }(a_{o_{1}}, \dots , a_{o_{m}}) \in \mathbf {F}$, let *R*^**D**^ contain the atom $R(b_{1}, \dots , b_{k})$ where, for 1 ≤ *α* ≤ *k*, we have $b_{\alpha } = a_{o_{j}}$ if *α* = *o*_*j*_ for some 1 ≤ *j* ≤ *m* and *b*_*α*_ = *d* if *α* = *i*_*j*_ for some 1 ≤ *j* ≤ *ℓ*. This completes the definition of **D**.

Finally, we define the mapping *μ* as *μ*(*x*) = *d* for all *x* ∈*fvar*(*branch*(*t*)).

With the description of the reduction complete, we claim that *μ* ∈ *p*(**D**) if and only if there is no homomorphism from **E** to **F**. We prove this property in two steps. First, we show that *μ* ∈ *p*(**D**) only depends on whether *μ* can be extended to *t* or not. After this we show that such an extension of *μ* exists if and only if there is a homomorphism $h \colon \mathbf {E} \rightarrow \mathbf {F}$.

First, observe that the only possible extension $\mu ^{\prime }$ of *μ* such that $\mu ^{\prime }(\tau ) \in \mathbf {D}$ for every *τ* ∈ *λ*(*branch*(*t*)) is $\mu ^{\prime }$ mapping every variable in *var*(*branch*(*t*)) to *d*. Moreover, for all nodes $t^{\prime \prime } \neq t$ in *ch*(*branch*(*t*)) the mapping $\mu ^{\prime }$ cannot be extended to $\lambda (t^{\prime \prime })$, since for all relation symbols *R* mentioned in $\lambda (t^{\prime \prime })$ we have *R*^**D**^ = *∅*. Thus, $\mu ^{\prime }$ is a pp-solution, and is a maximal pp-solution if and only if there exists no extension $\mu ^{\prime \prime }$ of $\mu ^{\prime }$ with $\mu ^{\prime \prime }(\tau ) \in \mathbf {D}$ for all *τ* ∈ *λ*(*t*).

Clearly, if $\mu ^{\prime }$ is a maximal pp-solution, then *μ* ∈ *p*(**D**). To see that *μ*∉*p*(**D**) if $\mu ^{\prime }$ is not a maximal pp-solution, assume the above mentioned extension $\mu ^{\prime \prime }$ of $\mu ^{\prime }$ exists. Then $\mu ^{\prime \prime }$ can be obviously extended to $\mu ^{\prime \prime \prime }$ with $\mu ^{\prime \prime \prime }(\tau ) \in \mathbf {D}$ for all $\tau \in \mathsf {cbranch}(t^{\prime })$ since for all atoms on $(\mathsf {cbranch}(t^{\prime }) \setminus \mathsf {cbranch}(t)) \cup \{t^{\prime }\}$, every possible atom over *dom*(**D**) is contained in **D**. Since $\mathsf {dom}(\mu ^{\prime \prime \prime })$ contains at least one free variable not in $\mathsf {dom}(\mu ^{\prime })$, this shows *μ*∉*p*(**D**).

It thus remains to show that the extension $\mu ^{\prime \prime }$ of $\mu ^{\prime }$ exists if and only if there is a homomorphism $h \colon \mathbf {E} \rightarrow \mathbf {F}$. To see that this is the case, observe that by construction every such homomorphism *h* in combination with $\mu ^{\prime }$ gives a homomorphism from **S** into **D**, and vice versa, every homomorphism $\mu \colon \mathbf {S} \rightarrow \mathbf {D}$ restricted to *dom*(**E**) gives the desired homomorphism. For the remaining atoms in *λ*(*t*) ∖**S**, observe that every possible mapping sends them into **D**, since **D** again contains every possible atom for these relations. □

To simplify the proof that tractability condition (b) is necessary, we first show that having bounded component width is a necessary condition on its own.

### **Lemma 3**

Let $\mathcal {P}$ be a decidable class of simple wdPTs of bounded arity. If there does not exist some constant *c* such that for every $p \in \mathcal {P}$ the component width is bounded by *c*, then p-Eval($\mathcal {P}$) is *coW*[1]-hard.

### *Proof*

The proof is an *FPT*-reduction from the problem of model checking FO sentences *ϕ*_*k*_ of the form $\phi _{k} = \forall x_{1} {\dots } \forall x_{k} \exists y \bigwedge _{i = 1}^{k} E_{i}(x_{i},y)$. Model checking for this class of sentences, parameterized by their size, is *W*[1]-hard [5]. Thus, consider a formula *ϕ*_*k*_ and a database **E**.

First, compute an arbitrary wdPT $p = (T, \lambda , \mathcal {X}) \in \mathcal {P}$ with a component width of at least *k*. Such a wdPT consists by assumption (otherwise the component width was bounded by *k*). W.l.o.g. we assume that *p* contains only binary atoms: Since we assume a bounded arity, binary atoms can be easily encoded into atoms of higher arity. Consider some relevant node *t* ∈ *T* and a node component $\mathbf {S} \in \mathcal {N}\mathcal {C}(t)$ such that the component width of **S** is at least *k* (they exist by construction). Since we assume relations to be of some bounded arity, **S** cannot be of type (1) (Definition 7). W.l.o.g. we thus assume that **S** is of type (2).

Since *t* is relevant, either for $t^{\prime } = t$ or some descendant $t^{\prime }$ of *t* we have $\mathsf {fvar}(t^{\prime }) \setminus \mathsf {fvar}(\mathsf {branch}(t^{\prime })) \neq \emptyset $. Choose one such $t^{\prime }$ at a minimal distance to *t*.

As in the proof of Lemma 2, for the description of **D** we assume for all *R*(**v**) ∈ *λ*(*T*) that **v** contains only variables. Recall that we are dealing with simple wdPTs, thus each relation symbol *R* occurs at most once within *λ*(*T*).

For each relation symbol *R* mentioned outside of $\lambda (\mathsf {cbranch}(t^{\prime }))$, let *R*^**D**^ = *∅*.

For each relation symbol *R* mentioned in $\lambda (\mathsf {cbranch}(t^{\prime })) \setminus \mathbf {S}$, let *ℓ* be the arity of *R* and *R*^**D**^ = {*R*(**v**)∣**v** ∈*dom*(**E**)^*ℓ*^}.

For the relation symbols *R* mentioned in **S**, proceed as follows. Choose *k* interface variables $v_{1}, \dots , v_{k} \in \mathcal {I}({\mathbf {S}}, t) $. Let *L* = *var*(**S**) ∖*var*(*branch*(*t*)) be the “local variables” of **S**. Observe that **S** being a node component of type (2) implies *L*≠*∅*. For the same reason, for each of the variables *v*_*i*_, there must exist at least one atom *R*_*i*_(*v*_*i*_, *z*_*i*_) or *R*_*i*_(*z*_*i*_, *v*_*i*_) for some *z*_*i*_ ∈ *L*. We will assume *R*_*i*_(*v*_*i*_, *z*_*i*_) in the following, the other case is analogous. Now for each *v*_*i*_, fix one such atom. Based on this, we define the following atoms to be contained in **D**:

For each of the selected atoms *R*_*i*_(*v*_*i*_, *z*_*i*_), let $R_{i}^{\mathbf {D}} = E_{i}^{\mathbf {E}}$, i.e., we let *R*_*i*_ simulate exactly *E*_*i*_. For every atom $R(z, z^{\prime }) \in \mathbf {S}$ such that $z, z^{\prime } \in L$, define *R*^**D**^ = {*R*(*d*, *d*)∣*d* ∈*dom*(**E**)}. For the remaining atoms $R(z, z^{\prime }) \in \mathbf {S}$, define *R*^**D**^ = {*R*(*a*, *b*)∣*a*, *b* ∈*dom*(**E**)}.

Finally, *μ* is an arbitrary mapping $\mathsf {fvar}(\mathsf {branch}(t)) \rightarrow \mathsf {dom}(\mathbf {E})$.

It now follows by the same arguments as in the proof of Lemma 2 that we have *μ*∉*p*(**D**) if and only if for every extension $\mu ^{\prime }$ of *μ* to *var*(*branch*(*t*)), there exists an extension *ν* of $\mu ^{\prime }$ such that *ν*(*τ*) ∈**D** for all *τ* ∈**S**.

To complete this proof, we thus only need to show that such an extension exists if and only if *ϕ*_*k*_ is satisfied. First, assume that *ϕ*_*k*_ is satisfied. Then, for all *z* ∈ *L*, define *ν*(*z*) to be the value of *y* in *ϕ*_*k*_. This clearly maps **S** into **D**. Next, assume that *ϕ*_*k*_ is not satisfied. Then there exists some assignment to $x_{1}, \dots , x_{k}$ such that no suitable value for *y* exists. Then for the mapping $\mu ^{\prime }$ assigning exactly those values to the selected interface variables $v_{1}, \dots , v_{k}$, there exists no extension of $\mu ^{\prime }$ to **S**. This is because *L* defines a connected component in the Gaifman graph and because the definition of **D** forces all variables in *L* that occur together in some atom in **S** to be mapped to the same value by $\mu ^{\prime }$. Thus, $\mu ^{\prime }$ has to map all “local variables” in **S** to the same value, which would provide a suitable value for *y*, leading to a contradiction. This concludes the proof. □

### **Lemma 4**

Let $\mathcal {P}$ be a decidable class of simple wdPTs of bounded arity that does not satisfy tractability condition (b). Then p-Eval($\mathcal {P}$) is either *coW*[1]- or *W*[1]-hard.

### *Proof*

First, assume that there exists some constant that is, for all $p \in \mathcal {P}$, an upper bound on the component width. Otherwise, p-Eval($\mathcal {P}$) is *coW*[1]-hard by Lemma 3. In particular, we may thus assume that all relations in all instances of $(\lambda (T^{\prime }) \cup \bigcup _{i=1}^{n} \{\mathsf {cia}({\mathbf {S}_{i}}) \}) \setminus \mathsf {fvar}(T^{\prime })$ for all $p \in \mathcal {P}$ are of bounded arity.

Let $\textit {solcheck}(\mathcal {P})$ be the class of all sets of atoms $(\lambda (T^{\prime }) \cup \bigcup _{i=1}^{n} \{\mathsf {cia}({\mathbf {S}_{i}}) \}) \setminus \mathsf {fvar}(T^{\prime })$ for $\mathcal {P}$ as defined in tractability condition (b). We reduce the problem $\mathrm {p\textup {-}HOM}(\textit {solcheck}(\mathcal {P}))$ to p-Eval($\mathcal {P}$) via an *FPT* reduction. The result then follows directly by Theorem 1, since if (b) is false then $\textit {solcheck}(\mathcal {P})$ has unbounded treewidth. The rest of this proof gives the desired reduction.

Let **E**, **F** be an instance of $\mathrm {p\textup {-}HOM}(\textit {solcheck}(\mathcal {P}))$. We construct a wdPT *p*, a database **D**, and a mapping *μ* such that *μ* ∈ *p*(**D**) if and only if there is a homomorphism from **E** to **F**.

First of all, find a $p = ((T,r), \lambda , \mathcal {X}) \in \mathcal {P}$ and a subtree $T^{\prime }$ of *T* containing *r* such that $\mathbf {E} = (\lambda (T^{\prime }) \cup \bigcup _{i=1}^{n} \{\mathsf {cia}(\mathbf {S}_{i})\}) \setminus \mathsf {fvar}(T^{\prime })$ for some combination $(\mathbf {S}_{1}, \dots , \mathbf {S}_{n}) \in \mathcal {N}\mathcal {C}({t_{1}}) \times {\dots } \times \mathcal {N}\mathcal {C}({t_{n}}) $ where $\{t_{1}, \dots , t_{n}\} = ch(T^{\prime }) \cap \mathsf {relv}(T)$. Such *p* exists by assumption and, since $\mathcal {P}$ is decidable, can be computed.

**Fig. 4:**
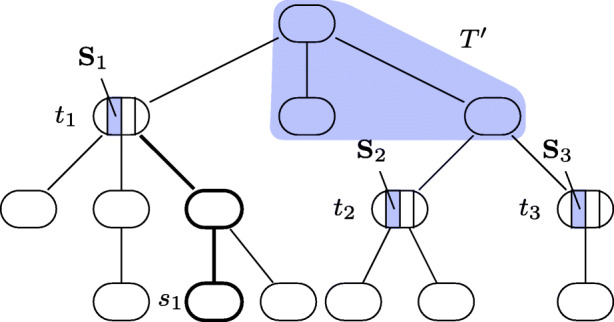
Illustration of the different parts of a wdPT distinguished in the proof of Lemma 4. The “slots” in the nodes *t*_1_, *t*_2_, and *t*_3_ represent the node components of these nodes. While we assume *t*_2_ and *t*_3_ to contain a free variable not occurring in $T^{\prime }$, *t*_1_ does not contain such a variable. The node *s*_1_ is one possible descendant of *t*_1_ with a free variable not in $T^{\prime }$

Next, the goal is to define a database **D** and a mapping *μ* such that *μ* ∈ *p*(**D**) if and only if a homomorphism from **E** to **F** exists. Before providing the formal construction, we sketch the idea first. Observe that **E** contains two types of atoms: $\lambda (T^{\prime })$ and the interface atoms *cia*(**S**_*i*_). The idea is to define **D** in such a way that there is a homomorphism $\mu ^{\prime } \colon \lambda (T^{\prime }) \rightarrow \mathbf {D}$ if and only if there exists $h \colon \lambda (T^{\prime }) \rightarrow \mathbf {F}$, and that $\mu ^{\prime }$ *cannot* be extended to any child node of $T^{\prime }$ if and only if *h* can be extended to all atoms *cia*(**S**_*i*_). Consider the depiction of a possible wdPT *p* in Fig. 4.

To implement the overall idea, we proceed as follows. We use atoms with the relation symbols in $\lambda (T^{\prime })$ to simulate **F**. For each child node *t*_*i*_ of $T^{\prime }$, we distinguish between the relation symbols in **S**_*i*_ and those not in **S**_*i*_. For those not in **S**_*i*_, we provide all possible atoms over the domain in **D**. I.e., every homomorphism on $\lambda (T^{\prime })$ can always be extended to the atoms not in **S**_*i*_. Whether it can be extended to all of *λ*(*t*_*i*_) thus only depends on **S**_*i*_. For those atoms we provide values that are only compatible with a homomorphism on $\lambda (T^{\prime })$ if this homomorphism cannot be extended to *cia*(**S**_*i*_). However, given a mapping *μ* on $\mathsf {fvar}(T^{\prime })$, for *μ*∉*p*(**D**) it is not sufficient that every homomorphism $\mu ^{\prime } \colon \lambda (T^{\prime }) \rightarrow \mathbf {D}$ can be extended to at least one child node of $T^{\prime }$: this child node must also contain a free variable not occurring in $T^{\prime }$. If this is not the case for some child *t*_*i*_, we pick one descendant *s*_*i*_ of *t*_*i*_ that contains a new free variable (since *t*_*i*_ is relevant, *s*_*i*_ exists), and for all relation symbols on the path from *t*_*i*_ to *s*_*i*_, we let **D** contain all possible atoms over the domain, thus making sure that if $\mu ^{\prime }$ can be extended to *t*_*i*_, it can also be extended all the way to *s*_*i*_.

We continue the formal definition of the reduction. To define a database **D** and a mapping *μ* such that *μ* ∈ *p*(**D**) if and only if a homomorphism from **E** to **F** exists, we need to define the following sets of nodes first. Let $K = ch(T^{\prime }) \cap \mathsf {relv}({T}) = \{t_{1}, \dots , t_{n}\}$. For each *t*_*i*_ ∈ *K*, we define the set *N*_*i*_ of nodes as follows. If *fvar*(*t*_*i*_) ∖*fvar*(*branch*(*t*_*i*_))≠*∅* (i.e., *t*_*i*_ contains some “new” free variable), then *N*_*i*_ = *∅*. Otherwise, let *s*_*i*_ ∈ *T* be a descendant of *t*_*i*_ such that *fvar*(*s*_*i*_) ∖*fvar*(*branch*(*t*_*i*_))≠*∅* and such that this property holds for no other node on the path from *t*_*i*_ to *s*_*i*_. Then *N*_*i*_ = *cbranch*(*s*_*i*_) ∖*cbranch*(*t*_*i*_). (E.g., in Fig. 4, *N*_1_ contains the two nodes with bold borders.) Finally, let $N = \bigcup _{i=1}^{n} N_{i}$. We can now describe the database **D**. While doing so, we implicitly assume that for all positions where an atom *R*(**v**) of *p* contains a constant, all the atoms in *R*^**D**^ contain the same constant as **v**. I.e., we describe only the values for “variable positions” of **v**. Recall that we are dealing with simple wdPTs, thus each relation symbol *R* occurs at most once within *λ*(*T*).

For all atoms $R(\mathbf {y}) \in \lambda (T) \setminus (\lambda (T^{\prime }) \cup \lambda (K) \cup \lambda (N))$, let *R*^**D**^ = *∅*, i.e., for all atoms neither in $T^{\prime }$ nor in any of the relevant child nodes of $T^{\prime }$ (or their extensions to some node with a “new” free variable), no matching values exist in the database.

For all atoms $R(\mathbf {y}) \in \lambda (T^{\prime })$, we want to use them to simulate in **D** the relations in **F**. Observe that for each such atom, there exists an atom $R^{\prime }(\mathbf {z}) \in \mathbf {E}$ that was derived from *R*(**y**) by removing the free variables $\mathsf {fvar}(T^{\prime })$. Thus, for each atom of the form $R^{\prime }(\mathbf {a})$ (i.e., atoms with relation symbol $R^{\prime }$) in **F**, we add one atom *R*(**b**) to *R*^**D**^ where **b** contains a fixed domain value *d* ∈*dom*(**F**) at all positions where **y** contains a free variable, and the value from **a** at those positions where the variable still occurs in $R^{\prime }(\mathbf {z}^{\prime })$. I.e., *R*^**D**^ is designed in such a way that all variables $x \in \mathsf {fvar}(T^{\prime })$ can only be mapped to *d*.

The definition for the atoms in *K* is more involved. Consider some *t*_*i*_ ∈ *K*. Let **v** contain the existential interface variables of the node component $\mathbf {S}_{i} \in \mathcal {N}\mathcal {C}(t_{i})$ selected for the construction of **E**, and assume *cia*(**S**_*i*_) = *R*_*cia*_(**v**).

For all atoms *R*(**y**) ∈ *λ*(*t*_*i*_) ∖**S**_*i*_, set *R*^**D**^ = {*R*(**a**)∣**a** ∈*dom*(**F**)^*k*^} where *k* is the arity of *R*. For the atoms in **S**_*i*_, we distinguish between **S**_*i*_ being of type (1) or of type (2).

If **S**_*i*_ is of type (1), i.e., **S**_*i*_ is of the form **S**_*i*_ = {*R*(**y**)} for some *R*(**y**) ∈ *λ*(*t*_*i*_), define $R^{\mathbf {D}} = \{R(\mathbf {a}) \mid \mathbf {a} \in \mathsf {dom}(\mathbf {F})^{k} \text { and } R_{\mathsf {cia}}(\mathbf {a}_{\mathbf {v}}) \notin {\mathbf {F}}\}$, where **a**_**v**_ is the restriction of **a** to those positions in **y** with variables from **v** (and thus not containing variables from $\mathsf {fvar}(T^{\prime })$).

If **S**_*i*_ is of type (2), we distinguish two types of variables: those that occur in $\mathcal {I}({\mathbf {S}_{i}}, t) $, and those that do not appear in any node $t^{\prime } \in \mathsf {branch}(t_{i})$. We call these latter variables *new* variables and use as their domain the set *dom*(**F**)^|**v**|^, i.e., the set of all possible assignments of values from **F** to the variables in **v** from *R*_*cia*_(**v**). We assume that the encoding of the assignments **a** ∈*dom*(**F**)^|**v**|^ is such that we can look up the value that is given to a variable *v*_*i*_ ∈**v** by **a**. For the remaining variables in *var*(**S**_*i*_), we will use values from *dom*(**F**). For each atom *R*(**y**) ∈**S**_*i*_, the values in *R*^**D**^ are defined as follows:

Let **z** = **y** ∩ (*var*(**S**_*i*_) ∖**v**) (because **S**_*i*_ is of type (2), $\mathbf {z} \setminus \mathsf {fvar}(T^{\prime }) \neq \emptyset $). Then *R*^**D**^ contains an atom for each tuple satisfying all of the following four properties.

1. All variables in $\mathsf {fvar}(T^{\prime })$ get the value *d*.
2. All the variables in $\mathbf {z} \setminus \mathsf {fvar}(T^{\prime })$ get assigned the same value. Denote this value by *a*, and recall that *a* represents an assignment **a** ∈*dom*(**F**)^|**v**|^.
3. For all *v*_*i*_ ∈**v** ∩**y**, the value of *v*_*i*_ is consistent with the vector **a** represented by *a*.
4. We have *R*_*cia*_(**a**)∉**F**.

Because their arity is assumed to be bounded, all these relations can be constructed in polynomial time. To conclude the definition of **D**, for all atoms *R*(**y**) ∈ *λ*(*N*), set *R*^**D**^ = {*R*(**a**)∣**a** ∈*dom*(**D**)^*k*^}, where *k* is the arity of *R* and *dom*(**D**) is implicitly defined to consist of all values mentioned in the definition of **D**.

Finally, let *μ* be the mapping defined on all variables $x \in \mathsf {fvar}(T^{\prime })$ as *μ*(*x*) = *d*. To prove *μ* ∈ *p*(**D**) if and only if homomorphism $\mathbf {E} \rightarrow \mathbf {F}$ exists, we first show the following claim.

### *Claim 1*

Let *R*_*cia*_(**v**) be an interface atom and **S**_*i*_ the corresponding node component. Then, for a mapping $\mu ^{\prime } \colon \mathbf {v} \rightarrow \mathsf {dom}(\mathbf {F})$, we have that $R_{\mathsf {cia}}(\mu ^{\prime }(\mathbf {v})) \in \mathbf {F}$ if and only if there is no extension $\mu ^{\prime \prime }$ of $\mu ^{\prime } \cup \mu $ to *var*(*λ*(*t*_*i*_)) that maps all atoms in **S**_*i*_ into **D** (since $\mathbf {S}_{i} \subseteq \lambda (t_{i})$, this implies that there exists no extension $\mu ^{\prime \prime }$ of $\mu ^{\prime } \cup \mu $ that maps *λ*(*t*_*i*_) into **D**).

### *Proof (of the Claim)*

For node components of type (1), the claim is immediate. So let us assume for the rest of the proof that **S**_*i*_ is of type (2).

First let $R_{\mathsf {cia}}(\mu ^{\prime }(\mathbf {v})) \in \mathbf {F}$. Let us assume an arbitrary extension $\mu ^{\prime \prime }$ of $\mu ^{\prime } \cup \mu $ to *var*(**S**_*i*_). If $\mu ^{\prime \prime }$ does not satisfy conditions 1., 2., and 3. for all *R*(**y**) ∈**S**_*i*_, then clearly for this particular atom there exists no atom in **D** to which it can be mapped by $\mu ^{\prime \prime }$. We may thus assume that $\mu ^{\prime \prime }$ satisfies the first three conditions for all atoms *R*(**y**) ∈**S**_*i*_. Then all variables in $\mathsf {var}(\mathbf {S}_{i}) \setminus (\mathbf {v} \cup \mathsf {fvar}(T^{\prime }))$ take the same value under $\mu ^{\prime \prime }$, and this value corresponds exactly to the tuple $\mu ^{\prime }(\mathbf {v})$. But then $\mu ^{\prime \prime }$ does not satisfy condition 4. for any *R*(**y**) ∈**S**_*i*_ since $R_{\mathsf {cia}}(\mu ^{\prime }(\mathbf {v})) \in R_{\mathsf {cia}}^{\mathbf {F}}$ by assumption. Thus, *R*^**D**^ does not contain any atom onto which $\mu ^{\prime \prime }$ could map *R*(**y**) and thus $\mu ^{\prime \prime }$ cannot exist which completes the first direction.

For the other direction, assume that no extension $\mu ^{\prime \prime }$ of $\mu ^{\prime } \cup \mu $ maps all atoms in **S**_*i*_ into **D**. Then this is in particular true for those assignments satisfying conditions 1., 2., and 3. Note that every such assignment maps all variables in $\mathbf {z} \setminus \mathsf {fvar}(T^{\prime })$ to the same value, representing a mapping on **v**. Also, $\mu ^{\prime \prime }|_{\mathbf {v}} = \mu ^{\prime }$. Since $\mu ^{\prime \prime }$ fails to map **S**_*i*_ into **D** because of 4., we get that $R_{\mathsf {cia}}(\mu ^{\prime }(\mathbf {v})) \in R_{\mathsf {cia}}^{\mathbf {F}}$, which completes the proof of the claim. □

We continue the proof that *μ* ∈ *p*(**D**) if and only if a homomorphism $\mathbf {E}\rightarrow \mathbf {F}$ exists. First observe that *μ* ∈ *p*(**D**) if and only if on the one hand there is an extension $\mu ^{\prime }$ of *μ* to $\mathsf {var}(T^{\prime })$ that maps all atoms in $\lambda (T^{\prime })$ into **D** (of course, in general every subtree $T^{\prime \prime }$ containing the root node of *T* with $\mathsf {fvar}(T^{\prime \prime }) = \mathsf {dom}(\mu )$ is a potential candidate, but given the construction of **D**, the subtree $T^{\prime }$ is the only possible candidate) and, on the other hand, for all *t*_*i*_ ∈ *K*, we have that there does not exist an extension of $\mu ^{\prime }$ onto *λ*(*t*_*i*_) ∪ *λ*(*N*_*i*_). (In fact, extending the mapping to any descendant of *t*_*i*_ that contains some additional free variable would work. However, the only nodes with non-empty relations in **D** are those mentioned in *N*.)

By the construction of **D** for atoms in *λ*(*N*), for every *t*_*i*_ ∈ *K* it follows immediately that there exists an extension of $\mu ^{\prime }$ onto *λ*(*t*_*i*_) ∪ *λ*(*N*_*i*_) if and only if there exists an extension to *λ*(*t*_*i*_). This is because for the atoms in *λ*(*N*) the database **D** contains all possible atoms, thus every extension $\mu ^{\prime \prime }$ of $\mu ^{\prime }$ onto *λ*(*t*_*i*_) can be further extended to all atoms in *λ*(*N*_*i*_).

Note that the existence of an extension of $\mu ^{\prime }$ onto *λ*(*t*_*i*_) is, by the Claim shown above, equivalent to $\mu ^{\prime }$ sending *R*_*cia*_(**v**) into **F**. So *μ* ∈ *p*(**D**) if and only if there is a homomorphism from **E** into **F**. This completes the proof. □

## Tractability Conditions for Non-simple wdPTs

As already mentioned at the beginning of Section 3, the tractability conditions presented there are not restricted to simple wdPTs, but also apply to arbitrary wdPTs. I.e., deciding p-Eval($\mathcal {P}$) is in *FPT* also for classes $\mathcal {P}$ of non-simple wdPTs. However, in case that the same relation symbol may occur in more than one atom throughout the query, the tractability criteria stated in the previous section miss important classes of tractable wdPTs. In the following, we discuss more general tractability notions.

The key difference for non-simple wdPTs is that in this setting, a set of atoms is not necessarily its own core. Since for the homomorphism problem, not the treewidth of the set of atoms, but the treewidth of its *core* determines the complexity of the problem as shown by Grohe [12] and recalled in Theorem 1, the concept of cores must also be taken into account for the tractability conditions. To do so, we revisit both, the notion of cores and the homomorphism problem, studying variations more suitable to our setting, and then apply these results to extend the definition of tractable classes.

### Extension Cores

In this section, we will introduce a variant of cores we call *extension core* that will turn out to be the right notion for wdPTs. The reason for this is that while the core is the suitable concept for the homomorphism problem (cf. Theorem 1), when evaluating wdPTs we actually deal with a variant of the homomorphism problem. To further motivate the idea of these extensions, recall that when deciding p-Eval($\mathcal {P}$) for some wdPT $p = (T, \lambda , \mathcal {X})$, one important step is to check, given some subtree $T^{\prime }$ of *T*, a mapping *μ* and some child node of $T^{\prime }$, whether $\mu ^{\prime }$ can be extended to this child. While this problem can be correctly stated as an instance of HOM, such a formulation could not adequately express all available information. In fact, compared to HOM, the problem at hand contains both, additional constraints (for some variables in the child node, the value is already determined by $\mu ^{\prime }$) and additional information (we know that $\mu ^{\prime }$ maps $\lambda (T^{\prime })$ into the database). The formulation as an instance of HOM could not utilize all of the information at hand, and as a result the problem might look harder than it actually is.

#### *Example 10*

**Fig. 5:**
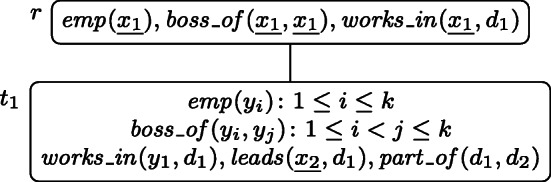
The wdPT *p*_*k*_ from Example 10. The free variables are underlined

Consider the (non-simple) wdPT *p*_*k*_ = (*T*, *λ*_*k*_,{*x*_1_, *x*_2_}) (parameterized by *k*) depicted in Fig. 5 consisting of two nodes. Clearly, the class $\mathcal {P} = \{p_{k} \mid k \geq 2\}$ of wdPTs does neither satisfy tractability condition (a) nor (b). In both cases, the reason for the violation is the clique of *boss*_*of*(*y*_*i*_, *y*_*j*_) atoms in *t*_1_, which resides in a single node component.

Nevertheless, deciding p-Eval($\mathcal {P}$) is in *FPT*. As mentioned, the reason for this is that tractability for the homomorphism problem depends on the treewidth of the core of the structure, and not on the treewidth of the structure.

That is, consider tractability condition (b) first. It ensures that in line 7 of Algorithm 1 the CQ can be evaluated efficiently. Now condition (b) is not satisfied because of the “subtree” of *T* which contains all of *T*. In this case, the resulting CQ *q*_*k*_ is $\textit {Ans}(x_{1},x_{2}) \leftarrow \lambda _{k}(T)$. However, given concrete values *a* and *b* for *x*_1_ and *x*_2_, the evaluation problem for the class of Boolean CQs $\{q_{k}(a,b) \mid p_{k} \in \mathcal {P}\}$ (where *q*_*k*_(*a*, *b*) is the query *q*_*k*_ with *x*_1_ and *x*_2_ in *λ*_*k*_(*T*) being replaced by *a* and *b*, respectively) is tractable. This is because *core*(*λ*_*k*_(*T*)) = {*emp*(*x*_1_), *boss*_*of*(*x*_1_, *x*_1_), *works*_*in*(*x*_1_, *d*_1_), *leads*(*x*_2_, *d*_1_), *part*_*of*(*d*_1_, *d*_2_)} due to the homomorphism *h* with *h*(*y*_*i*_) = *x*_1_ for 1 ≤ *i* ≤ *k*. The treewidth of these cores is obviously bounded by some constant, and thus deciding the problem is tractable (c.f. Theorem 1).

While adapting tractability condition (b) is rather straightforward, the situation is more interesting for condition (a). Recall that the idea of condition (a) is to ensure that deciding whether a mapping on the interface variables of a node component can be extended to this node component is tractable. Consider the node component **S** in *t*_1_ that contains the set of *boss*_*of*(*y*_*i*_, *y*_*j*_) atoms. Clearly, for increasing *k* the treewidth of this component is not bounded, and neither is the treewidth of its core (since it already is its own core). Deciding whether a mapping on *d*_1_ (the only interface variable) can be extended to this component is nevertheless tractable.

The reason for this is that it is only necessary to consider mappings *μ* on *d*_1_ that have an extension which maps *λ*_*k*_(*r*) into a given database. Thus, for deciding the extension to *t*_1_, we can assume the existence of such a mapping on *λ*_*k*_(*r*). In our case, we thus know that the database contains a target for *emp*(*x*_1_), *boss*_*of*(*x*_1_, *x*_1_), and *works*_*in*(*x*_1_, *d*_1_). Thus, instead of considering just the core of **S**, we can consider the core of **S** ∪ *λ*_*k*_(*r*). Again by the same homomorphism as before, that is *h*(*y*_*i*_) = *x*_1_ for all 1 ≤ *i* ≤ *k*, we can now fold parts of **S** into *λ*_*k*_(*r*). In the next step, we can remove again all atoms from *λ*_*k*_(*r*), since for them the existence of a mapping into the database is already known, and finally only need to decide the existence of an extension of *μ* on the remaining set of atoms, which again, is tractable for $\mathcal {P}$.

To formalize this idea of “folding” parts of node components into the set of atoms on the branch to this component, we introduce the notion of an *extension core*. We then introduce the problem EXT($\mathcal {C}$) which captures the idea of finding extensions to a given mapping, and characterize its tractable classes. Using this problem, we then introduce refined versions of tractability conditions (a) and (b) based on the notion of the extension core, and show that these improved conditions indeed guarantee tractability for p-Eval($\mathcal {P}$).

Towards the definition of the *extension core*, for a set $\mathcal {A} \subseteq {\mathcal Const} \cup {\mathcal Var}$ of elements, let $\mathbf {Fix}_{\mathcal {A}}$ be the set of atoms $\mathbf {Fix}_{\mathcal {A}} = \{R_{a}(a) \mid a \in \mathcal {A}\}$ where each *R*_*a*_ is a unique relation symbol.

#### **Definition 10** (Extension Core)

Let (**A**, **B**) be a pair of sets of atoms. The *extension coreextcore*(**A**, **B**) is the set *extcore*(**A**, **B**) = (*core*(**A** ∪**B** ∪**Fix**_*dom*(**A**)_) ∖**Fix**_*dom*(**A**)_) ∖*dom*(**A**).

Said differently, the extension core is constructed by introducing a new relation for every domain element in **A** and then computing the core. That is, the extension core accounts on the one hand for the possibility that parts of **B** might be folded into **A** (and thus the extension of the homomorphism to these parts is guaranteed), and on the other hand for the fact that the mapping on *dom*(**A**) is fixed. Removing *dom*(**A**) is then possible because the mapping is already provided for these values.

#### *Example 11*

To illustrate Definition 10, consider the wdPT from Fig. 5, and let **A** = *λ*(*r*) and **B** = *λ*(*t*_1_). Then $\mathbf {Fix}_{\mathsf {dom}(\mathbf {A})} = \{R_{x_{1}}(x_{1}), R_{d_{1}}(d_{1})\}$. Then *core*(**A** ∪**B** ∪**Fix**_*dom*(**A**)_) = {*emp*(*x*_1_), *boss*_*of*(*x*_1_, *x*_1_), *works*_*in*(*x*_1_, *d*_1_), *leads*(*x*_2_, *d*_1_), *part*_*of*(*d*_1_, *d*_2_), $R_{x_{1}}(x_{1})$, $R_{d_{1}}(d_{1})\}$ (mapping all *y*_*i*_ to *x*_1_). The extension core then is $\{ \textit {emp}^{1=x_{1}}()$, $\textit {boss}\_{\textit {of}}^{1=x_{1},2=x_{1}}()$, $\textit {works}\_{\textit {in}}^{1=x_{1},2=d_{1}}()$, $\textit {leads}^{2=d_{1}}({x_{2}})$, $\textit {part}\_{\textit {of}}^{1=d_{1}}(d_{2})\}$.

### The Extension Problem

A task within the evaluation problem of wdPTs is to test whether a mapping on some subtree is maximal or can be extended to some child node. We formalize this by the following problem, where $\mathcal {C}$ is a class of pairs of sets of atoms. 
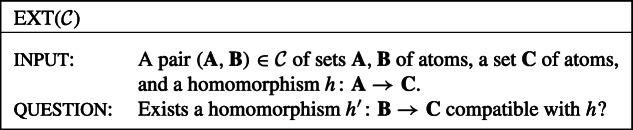


The parameterized problem p-EXT($\mathcal {C}$) is the problem EXT($\mathcal {C}$) parameterized by the size of (**A**, **B**).

The main difference between HOM and EXT is that in addition to a set of atoms, the input of EXT gets another set of atoms and a homomorphism that is already guaranteed to map this additional set of atoms into the target. When looking for tractable classes, this additional input has to be taken into account. This is exactly the role of extension cores as defined in the last section.

While here we use the extension problem to define tractable fragments of the evaluation problem of wdPTs with projection, we note that it also allows for an alternative formulation of the tractable classes of projection free wdPTs defined by Romero [24].

With these definitions settled, we next use extension cores to provide an exact characterization of the tractable classes $\mathcal {C}$ of the extension problem EXT($\mathcal {C}$). To this end, we define the treewidth of $\mathsf {extcore}(\mathcal {C})$ to be the maximum of the treewidth of *extcore*(**A**, **B**) for $(\mathbf {A}, \mathbf {B})\in \mathcal {C}$ if this maximum exists and $\infty $ otherwise.

#### **Theorem 3**

Assume that *FPT*≠*W*[1] and let $\mathcal {C}$ be a decidable class of pairs of sets of atoms. Then the following statements are equivalent:

1. The treewidth of $\mathsf {extcore}(\mathcal {C})$ is bounded by a constant.
2. The problem EXT($\mathcal {C}$) is in *PTIME*.
3. The problem p-E XT($\mathcal {C}$) is in *FPT*.

Theorem 3 is shown using a sequence of lemmas given below. First, we state an easy but important observation that we use tacitly throughout this section.

#### **Observation 4**

- Extension cores are unique up to isomorphism.
- For all sets **A**, **B** of atoms, we have *core*(*extcore*(**A**, **B**)) = *extcore*(**A**, **B**).

The first lemma in the sequence describes a crucial property of extension cores that will be used several times throughout the remainder of this section.

#### **Lemma 5**

An instance (**A**, **B**),**D**, *h* of EXT is a positive instance of EXT if and only if there exists a homomorphism $h^{\prime } \colon (\mathbf {A} \cup \mathbf {E}) \rightarrow \mathbf {D}$ that extends *h*, where **E** is the set *core*(**A** ∪**B** ∪**Fix**_*dom*(**A**)_) ∖**Fix**_*dom*(**A**)_ of atoms from the definition of extension cores.

#### *Proof*

Solving the instance (**A**, **B**),**D**, *h* of EXT is equivalent to solving the instance ((**A** ∪**B** ∪**Fix**_*dom*(**A**)_),(**D** ∪ *h*(**Fix**_*dom*(**A**)_)) of HOM (where *h*(**Fix**_*dom*(**A**)_) denotes the set **Fix**_*dom*(**A**)_ of atoms where all elements *a* ∈*dom*(*h*) are replaced by *h*(*a*)). This in turn is equivalent to deciding the existence of a homomorphism $h^{\prime } \colon (\mathbf {A} \cup \mathbf {E}) \rightarrow \mathbf {D}$ extending *h*. □

Next, we show the positive result, i.e., that the problem EXT($\mathcal {C}$) can be solved efficiently if the treewidth of the extension cores in $\mathcal {C}$ is bounded.

#### **Lemma 6**

Let $\mathcal {C}$ be a class of pairs of sets of atoms such that the treewidth of *extcore*(**A**, **B**) for all $(\mathbf {A}, \mathbf {B}) \in \mathcal {C}$ is bounded by some constant *c*. Then EXT($\mathcal {C}$) is in *PTIME*.

The proof of this result heavily relies on the notion of restricting a set **A** of atoms to a subset of its domain $\mathsf {dom}(\mathbf {A}) \setminus \mathcal {A}$ for $\mathcal {A} \subseteq \mathsf {dom}(\mathbf {A})$ already introduced in Section 2. Another important concept is the *projection of a pair*(**A**, **B**) *of sets of atoms under a mapping* *h*, where $\mathsf {dom}(h) \subseteq \mathsf {dom}(\mathbf {A})$, and the range of *h* is (some subset of) *dom*(**B**). This describes the process of first replacing in **A** all values *a* ∈*dom*(*h*) by *h*(*a*), and then, for the resulting structure $\mathbf {A}^{\prime }$, computing $\mathbf {A}^{\prime } \setminus \mathsf {dom}(\mathbf {B})$ (w.l.o.g. assuming *dom*(**A**) ∩*dom*(**B**) = *∅*). In the second step, for every new relation symbol *R*^*s*^ in $\mathbf {A}^{\prime } \setminus \mathsf {dom}(\mathbf {B})$ created from some relation symbol *R*, and every atom *R*(**a**) ∈**B** such that **a** is consistent with the values encoded in *s* (recall that *s* records the positions and their values that were removed from *R* when creating *R*^*s*^), we add an atom *R*^*s*^(**b**) to **B**, where **b** is the projection of **a** to the remaining positions in *R*^*s*^.

Both, restricting a set of atoms and the projection of pairs of atoms are well-known techniques. However, they occur in slightly different interpretations throughout the literature. Thus, to avoid any ambiguities, we provide full formal definitions for both of them. Being very technical definitions for common notions, they are given in the Appnedix.

The following observation not only is an immediate consequence of the above definitions, but also describes the intuition behind projecting a pair of sets of atoms and will be used in the proof of Lemma 6.

#### **Observation 5**

Let two sets **Q**, **D** of atoms over a relational schema *σ* and a mapping $h \colon \mathcal {Q} \rightarrow \mathsf {dom}(\mathbf {D})$ be given where $\mathcal {Q} \subseteq \mathsf {dom}(\mathbf {Q})$, and let $\mathbf {Q}^{\prime }, \mathbf {D}^{\prime }$ be the projection of (**Q**, **D**) under *h*. Then there exists some extension $h^{\prime } \colon \mathbf {Q} \rightarrow \mathbf {D}$ of *h* if and only if there exists a homomorphism $\hat h\colon \mathbf {Q}^{\prime } \rightarrow \mathbf {D}^{\prime }$ (i.e., if $(\mathbf {Q}^{\prime }, \mathbf {D}^{\prime })$ is a positive instance of HOM).

We now have everything in place to prove Lemma 6.

#### *Proof (of Lemma 6)*

Let $(\mathbf {A}, \mathbf {B}) \in \mathcal {C}$, **D**, and *h* be an instance of EXT($\mathcal {C}$). By Lemma 5, this problem is equivalent to asking for the existence of a homomorphism $h^{\prime } \colon (\mathbf {A} \cup \mathbf {E}) \rightarrow \mathbf {D}$ that extends *h*, where $\mathbf {E} = \mathsf {core}(\mathbf {A} \cup \mathbf {B} \cup \mathbf {Fix}_{\mathcal {A}}) \setminus \mathbf {Fix}_{\mathcal {A}}$ and $\mathcal {A} = \mathsf {dom}(\mathbf {A})$. By Observation 5, this is equivalent to the instance $((\mathbf {A} \cup \mathbf {E})', \mathbf {D}^{\prime })$ of HOM, where $((\mathbf {A} \cup \mathbf {E})', \mathbf {D}^{\prime })$ is the projection of ((**A** ∪**E**),**D**) under *h*.

For **EXT** = *extcore*(**A**, **B**) and **F** = **D**, we next show that $((\mathbf {A} \cup \mathbf {E})^{\prime }, \mathbf {D}^{\prime }) = (\mathbf {EXT}^{\prime }, \mathbf {F}^{\prime })$ where $(\mathbf {EXT}^{\prime }, \mathbf {F}^{\prime })$ is the projection of (**EXT**, **F**) under *h*. First of all, observe that $\mathbf {D}^{\prime } = \mathbf {F}^{\prime }$ does not necessarily hold, since the content of $\mathbf {D}^{\prime }$ and $\mathbf {F}^{\prime }$ depends on the result of the projection with $(\mathbf {A} \cup \mathbf {E})^{\prime }$ and $\mathbf {EXT}^{\prime }$, respectively. However, if these left hand sides coincide, the equality $\mathbf {D}^{\prime } = \mathbf {F}^{\prime }$ obviously holds.

We thus show that $(\mathbf {A} \cup \mathbf {E})^{\prime } = \mathbf {EXT}^{\prime }$. First of all, we have that $\mathbf {A} \cup \mathbf {E} = \mathbf {A} \cup \Big (\mathsf {core}\big (\mathbf {A} \cup \mathbf {B} \cup \mathbf {Fix}_{\mathcal {A}}\big ) \setminus \mathbf {Fix}_{\mathcal {A}}\Big ) \subseteq (\mathbf {A} \cup \mathbf {B})$. Moreover, since $h \colon \mathbf {A} \rightarrow \mathbf {D}$, when computing the projection under *h* we drop all atoms from *A*, and therefore $(\mathbf {A} \cup \mathbf {E})^{\prime }$ does not contain any atoms derived from **A**. It is thus safe to conclude that $(\mathbf {A} \cup \mathbf {E})^{\prime } = \mathbf {E}^{\prime }$, where $(\mathbf {E}^{\prime }, \mathbf {D}^{\prime })$ is the projection of (**E**, **D**) under *h*.

Next, recall that $\mathsf {extcore}(\mathbf {A}, \mathbf {B}) = \mathbf {E} \setminus \mathcal {A}$. Thus, the only difference between $\mathbf {EXT}^{\prime } = (\mathbf {E} \setminus \mathcal {A})^{\prime }$ and $(\mathbf {A} \cup \mathbf {E})^{\prime } = \mathbf {E}^{\prime }$ is that in the first case, the restriction of **E** under $\mathcal {A}$ is computed, before the projection under *h*. However, since $\mathsf {dom}(h) = \mathsf {dom}(\mathbf {A}) = \mathcal {A}$, it can be easily checked that this results in the same structures, and thus $\mathbf {EXT}^{\prime } = (\mathbf {A} \cup \mathbf {E})^{\prime }$.

Now the treewidth of **EXT** is bounded by *c*, and therefore also the treewidth of $\mathbf {EXT}^{\prime }$ (taking subgraphs does not increase the treewidth). As a result, the existence of a homomorphism $\mathbf {EXT}^{\prime } \rightarrow \mathbf {F}^{\prime }$ can be decided in polynomial time [7], which proves the lemma. □

With this showing the upper bounds in Theorem 3, we turn towards the lower bound, for which the following property will turn out to be important.

#### **Lemma 7**

Let **A** and **B** be sets of atoms and let **C** be the set of atoms *core*(**A** ∪**B** ∪**Fix**_*dom*(**A**)_) from the definition of the extension core. If *h* is a homomorphism from **C** to itself, then *h* is bijective.

#### *Proof*

Since **C** is, by definition, a core, any homomorphism from **C** to itself is an isomorphism. □

The next result shows that the lower bounds in Theorem 3 are optimal by using the characterization of tractable classes (for both, *PTIME* and *FPT*) of $\mathrm {p\textup {-}HOM}(\mathcal {C})$ provided by Grohe [12].

#### **Lemma 8**

Let $\mathcal {C}$ be a decidable class of pairs of sets of atoms and let $\mathsf {extcore}(\mathcal {C})$ be the class of extension cores of the pairs in $\mathcal {C}$. Then $\mathrm {p\textup {-}HOM}(\mathsf {extcore}(\mathcal {C}))$≤_*FPT*_ p-EXT($\mathcal {C}$).

#### *Proof*

Let $\mathcal {C}$ be a decidable class of pairs of sets of atoms, and let (**L**, **T**) be an instance of $\mathrm {p\textup {-}HOM}(\mathsf {extcore}(\mathcal {C}))$. We reduce this problem to an instance of p-EXT($\mathcal {C}$).

Borrowing from the notation of databases introduced in Section 2, for an arbitrary set **A** of atoms and a relation symbol *R*, throughout this proof we write *R*^**A**^ to denote the set of all atoms in **A** with relation symbol *R*.

In the first step, we compute some pair $(\mathbf {A}, \mathbf {B}) \in \mathcal {C}$ such that *extcore*(**A**, **B**) = **L**. By assumption, such a pair exists and, because $\mathcal {C}$ is decidable, can be computed. Next, we need to show that there exists a set **D** of atoms and a homomorphism $h \colon \mathbf {A} \rightarrow \mathbf {D}$ such that there exists a homomorphism $h^{\prime } \colon (\mathbf {A} \cup \mathbf {B}) \rightarrow \mathbf {D}$ that is an extension of *h* if and only if there exists a homomorphism from **L** to **T**. However, by utilizing Lemma 5, we will work in a slightly different setting.

Let **E** be the set of atoms *core*(**A** ∪**B** ∪**Fix**_*dom*(**A**)_) ∖**Fix**_*dom*(**A**)_ from the definition of extension cores. By Lemma 5, the desired extension $h^{\prime }$ of *h* exists if and only if there exists a homomorphism $\hat h \colon (\mathbf {A} \cup \mathbf {E}) \rightarrow \mathbf {D}$ that extends *h*. Observe that the set **D** and homomorphism *h* are still the same as above. We will work in the latter setting with **E** instead of **B** as this turns out to be easier.

We define the set **D** of atoms over the same schema as **E** as follows:

- The domain *dom*(**D**) = *dom*(**T**) ×*dom*(**E**), i.e., the elements represent pairs of elements from **T** and **E**, respectively.
- For each relation symbol *R* of arity *k*, and every $R(a_{1}, \dots , a_{k}) \in {\mathbf {E}}$, the set **D** contains the following atoms:
  Let $\{i_{1}, \dots , i_{\ell }\} \subseteq \{1, \dots , k\}$ be all those positions of $(a_{1}, \dots , a_{k})$ such that $a_{i_{j}} \in \mathsf {dom}(\mathbf {A})$, and let $\{o_{1}, \dots , o_{m}\} = \{1, \dots , k\} \setminus \{i_{1}, \dots , i_{\ell }\}$ be all those positions such that $a_{o_{j}} \notin \mathsf {dom}(\mathbf {A})$, i.e., $a_{o_{j}} \in \mathsf {dom}(\mathbf {B}) \setminus \mathsf {dom}(\mathbf {A})$. Let furthermore $R^{\prime }$ be the relation symbol derived for $R(a_{1}, \dots , a_{k}) \in {\mathbf {E}}$ when computing the projection **E** ∖*dom*(**A**) = *extcore*(**A**, **B**).
  Now, for every pair (**d**_1_, **d**_2_) of tuples $\mathbf {d}_{1} = (d_{o_{1}}, \dots , d_{o_{m}})$ with $R^{\prime }(\mathbf {d}_{1}) \in \mathbf T$ and $\mathbf {d}_{2} = (d_{i_{1}}, \dots , d_{i_{\ell }}) \in \mathsf {dom}(\mathbf T)^{\ell }$, add the atom $R((d_{1}, a_{1}), \dots , (d_{k},a_{k}))$ to **D**. (Observe that by slight abuse of notation, in order to simplify the description we denote the positions in **d**_1_ and **d**_2_ according to the position in *R* they originate from.) Thus, intuitively, we replace all domain elements from *dom*(**A**) with all possible combinations of elements from *dom*(**T**).
  These are all the tuples in **D**.

It is worth pointing out that in case $R^{\prime }$ is not part of the schema of **T** or $(R^{\prime })^{\mathbf T}$ is empty, then by this definition also *R*^**D**^ is empty. The resulting instance will therefore be a simple “no” instance, because *R*^**E**^ is non-empty. However, in this case we also have that $(R^{\prime })^{\mathbf {L}}$ is nonempty, and therefore also (**L**, **T**) is a trivial “no” instance.

Finally, we define the mapping $h \colon \mathsf {dom}(\mathbf {A}) \rightarrow \mathsf {dom}(\mathbf {D})$ as *h*(*a*) = (*d*, *a*) for some arbitrary but fixed element *d* ∈*dom*(**T**). Since **D** contains one atom $R((d_{1},a_{1}), \dots , (d_{k},a_{k}))$ for every atom $R(a_{1}, \dots , a_{k}) \in \mathbf {A}$ and every combination of $d_{1}, \dots , d_{k} \in \mathsf {dom}(\mathbf T)$, clearly *h* is a homomorphism $h \colon \mathbf {A} \rightarrow \mathbf {D}$.

It remains to prove that there indeed exists a homomorphism $g \colon \mathbf {L} \rightarrow \mathbf T$ if and only if *h* can be extended to a homomorphism $\hat h \colon \mathbf {E} \rightarrow \mathbf {D}$.

First assume that *g* exists. Then define an extension $\hat h$ of *h* to *dom*(**E**) as $\hat h(a) = (g(a),a)$ for all *a* ∈*dom*(**E**) ∖*dom*(**A**). The mapping *g* is indeed defined on all these elements, since *dom*(**E**) ∖*dom*(**A**) = *dom*(*extcore*(**A**, **B**)) = *dom*(**L**) because *extcore*(**A**, **B**) = **L**. For *a* ∈*dom*(**A**) we need not define $\hat h$ since *h* is already defined on these elements, and $\hat h$ extends *h*. It now follows immediately from the construction of **D** that $\hat h$ is indeed the required homomorphism.

For the other direction, assume that $\hat h$ exists. First, observe that **D** projected onto the second component of its domain elements gives **E**. Thus, $\hat h$ is a bijection in this second coordinate by Lemma 7. Let *π*_2_ be the projection to the second coordinate. Then *π*_2_ ∘ *h* is an automorphism of **E**, and thus there is a $n \in \mathbb {N}$ such that (*π*_2_ ∘ *h*)^*n*^ = *id* (where *id* denotes the identity mapping). Consequently, w.l.o.g. we assume that *π*_2_ ∘ *h* = *id*. For every *a* ∈*dom*(**L**) = *dom*(*extcore*(**A**, **B**)) define *g*(*a*) to be the value *d* such that $\hat h(a) = (d,a)$. Then again by definition of **D** it follows immediately that for all relation symbols *R* and tuples **a** ∈ *R*^**L**^ we have *g*(**a**) ∈ *R*^**T**^.

Observing that all constructions can be done efficiently completes the proof. □

Theorem 3 now follows immediately. (1) ⇒ (2) follows from Lemma 6. The implication (2) ⇒ (3) follows immediately. Finally, if the treewidth of $\mathsf {extcore}(\mathcal {C})$ is not bounded, then $\mathrm {p\textup {-}HOM}(\mathsf {extcore}(\mathcal {C}))$ is not in *FPT* by Grohe [12]. Thus, by Lemma 8, the problem p-EXT($\mathcal {C}$) is not in *FPT*, which shows (3) ⇒ (1).

### Tractability Conditions for Arbitrary wdPTs

With the notion of extension cores, we now have a tool to adapt tractability conditions (a) and (b) to also account for the core of sets of atoms, and to make use of the knowledge that when looking for extensions, the existence of certain mappings can be assumed. Recall the intuition described at the beginning of Section 6.1 and Example 10. In this situation, the maximality test towards a single node component can be easily expressed as an instance of EXT($\mathcal {C}$). More precisely, given a wdPT $(T, \lambda , \mathcal {X})$, a database **D**, a subtree $T^{\prime }$ of *T*, and a node component $\mathbf {S} \in \mathcal {N}\mathcal {C}({t}) $ for some node *t* ∈ *ch*(*T*), testing if some mapping $\mu ^{\prime }$ can be extended to **S** is the instance $(\lambda (T^{\prime }), \mathbf {S}), \mathbf {D}, \mu $ of EXT($\mathcal {C}$). One way to adapt tractability condition (a) – recall that intuitively this is the condition ensuring that the test for maximality is tractable – would thus be to associate with each class $\mathcal {P}$ of wdPTs a class $\mathcal {C}$ of all relevant pairs $(\lambda (T^{\prime }), \mathbf {S})$, and to require EXT($\mathcal {C}$) to be in *FPT*. However, using the easy characterization of Theorem 3, we state the refined variant of tractability condition (a) directly in terms of extension cores. 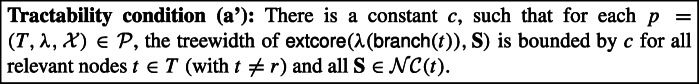


Three comments are in order. First, observe that for the case of simple wdPTs, tractability condition (a’) is equivalent to tractability condition (a). Second, note that unlike in the above discussion, condition (a’) mentions *branch*(*t*) instead of $T^{\prime }$. This is because the condition has to be satisfied for all subtrees $T^{\prime }$ with $t \in ch(T^{\prime })$. Among these, *branch*(*t*) is the minimal one. Thus, for all subtrees containing *branch*(*t*), the treewidth of $\mathsf {extcore}(\lambda (T^{\prime }), \mathbf {S})$ is at most the treewidth of *extcore*(*λ*(*branch*(*t*)),**S**). Third, recall that the intuition used above for motivating tractability condition (a’) does not match the actual idea implemented in Algorithm 1. In fact, not testing maximality for one possible mapping on subtrees $T^{\prime }$ after the other, but merging this test with finding mappings on the existential variables in $T^{\prime }$ was actually a crucial step in the development of the algorithm. We will later describe the necessary changes to the algorithm in order for it to utilize the additional information given by the existence of a mapping that maps $\lambda (T^{\prime })$ into the database, but first we reconsider tractability condition (b).

Recall that tractability condition (b) ensures that the CQs for finding suitable, maximal mappings on the existential variables are tractable. By Grohe [12], this is the case if and only if the core of the CQs has bounded treewidth. However, to account for the fact that for some free variables a mapping was provided as part of the input, when computing the core, these variables must be mapped onto themselves. This requirement is again naturally expressed in terms of extension cores. 
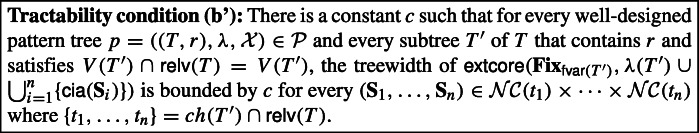


Analogously to tractability condition (a’), for simple wdPTs tractability condition (b’) is equivalent to condition (b). However, there exist classes of non-simple wdPTs that satisfy conditions (a’) and (b’), but not (a) and (b). An example for such a class of wdPTs was described in Example 10. Also, the two tractability conditions are independent of each other, as illustrated by the following example.

#### *Example 12*

For a class of wdPTs that satisfies condition (a’) but not (b’), consider the class of wdPTs containing the wdPTs from Fig. 3 for all *k* ≥ 1. For any subtree containing the node *t*_2_, condition (b’) is not satisfied because of the *k*-clique of *yclique*_*ij*_(*y*_*i*_, *y*_*j*_) atoms. However, condition (a’) is satisfied, since all variables in this clique are interface variables.

For the opposite case, recall the wdPT in Fig. 5, but assume that the atom *emp*(*y*_1_) was part of *λ*(*r*) instead of *λ*(*t*_1_). Clearly, the core of *λ*(*T*) remains unchanged, and thus of bounded treewidth. Also the set of atoms *boss*_*of* remains part of a single node component **S**. Now when computing the extension core of (*λ*(*r*),**S**), *y*_1_ is part of *λ*(*r*), and thus must be mapped onto itself. As a result, all *y*_*i*_ must be mapped onto themselves. After removing the variables from *λ*(*r*), a (*k* − 1)-clique remains, and thus condition (a’) is not satisfied.

Conditions (a’) and (b’) therefore describe a proper extended set of classes of wdPTs. Next, we discuss why query evaluation is indeed tractable for these classes.

In fact, Algorithm 1 already describes a correct *FPT* algorithm even for classes of arbitrary wdPTs satisfying conditions (a’) and (b’). The only change necessary does not happen directly within Algorithm 1, but in the computation of *stop*(**S**, **D**) (for some node component **S** and database **D** in line 6). So far, given an instance *p*, **D**, *μ* of p-Eval($\mathcal {P}$), the content of *stop*(**S**, **D**) was computed as follows: for every possible mapping *ν* on $\mathcal {I}({\mathbf {S}}, t) $ compatible with *μ*, check if it can be extended to a homomorphism $\nu ^{\prime } \colon \mathbf {S} \rightarrow \mathbf {D}$. If this is not the case, include $\nu _{|\mathcal {I}^{\exists }({\mathbf {S}}, t) }$ in *stop*(**S**, **D**). The only change is that instead of testing for a homomorphism from **S** into **D**, we now decide whether to add the tuple into *stop*(**S**, **D**) based on the non-existence of a homomorphism $h^{\prime } \colon \mathsf {extcore}(\lambda (\mathsf {branch}(t)), \mathbf {S}) \rightarrow \mathbf {D}$ extending *h*. I.e., formulated in terms of the problem EXT($\mathcal {C}$), we have $h_{|\mathcal {I}^{\exists }(\mathbf {S}, t)} \in \textit {stop}(\mathbf {S}, \mathbf {D})$ if (*∅*, *extcore*(*λ*(*branch*(*t*)),**S**)),*h*, **D** is a negative instance of EXT($\mathcal {C}$).

Note that this new definition does not necessarily give the same sets *stop*(**S**, **D**) as we would get under the original definition, as demonstrated by the following very simple example.

#### *Example 13*

**Fig. 6:**
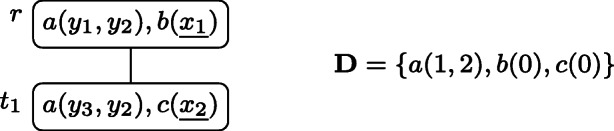
The wdPT *p* and database instance **D** from Example 13. The free variables are underlined

Consider the wdPTs *p* as depicted in Fig. 6. For the node component **S** = {*a*(*y*_3_, *y*_2_)} in *t*_1_, we get *cia*(**S**) = *R*(*y*_2_), and *extcore*(*λ*(*r*),**S**) = *∅*. Then, following the definition in Section 4, we get *stop*(**S**, **D**) = {*R*(0),*R*(1)} since mapping *y*_2_ to either of these values does not map *a*(*y*_3_, *y*_2_) into **D**. In contrast, when working with *extcore*(*λ*(*r*),**S**), we get *stop*(**S**, **D**) = *∅*.

The reason for this is that replacing **S** by *extcore*(*λ*(*branch*(*t*)),**S**) implies that the mapping *h* that shall be extended to **S** is (or can be extended to) a homomorphism $\mathsf {branch}(t) \rightarrow \mathbf {D}$. Obviously, in the example this is not the case for any mapping that maps *y*_2_ to either 0 or 1.

As we will show below, this difference has no effect on the output of Algorithm 1, since the additional values in *stop*(**S**, **D**) according to the original definition are never involved in any solution anyway.

By combining all of this, we get the following result as a proper extension of Lemma 1.

#### **Theorem 6**

Let $\mathcal {P}$ be a decidable class of wdPTs. If tractability conditions (a’) and (b’) hold for $\mathcal {P}$, then p-Eval($\mathcal {P}$) can be solved in *FPT*.

#### *Proof*

First of all, observe that under these conditions Algorithm 1 is still in *FPT*: with the exception of evaluating the query *q* (line 7) and computing the set *stop*(**S**, **D**) (line 6), all the arguments from Section 4 still apply.

For deciding $\mu (\mathbf {x}) \in q(\mathbf {D}^{\prime })$ in line 7, observe that this is equivalent to deciding the instance $(\emptyset , \lambda (T^{\prime }) \cup \{ \mathsf {cia}({\mathbf {S}_{1}}) , \dots , \mathsf {cia}(\mathbf {S}_{n})\}), \mu , \mathbf {D}^{\prime }$ of EXT($\mathcal {C}$), which is in *FPT* because of Theorem 3 and the fact that tractability condition (b’) is satisfied.

For computing *stop*(**S**, **D**), first of all observe that since the component interface atoms contain unique relation symbols and no variables from $\mathsf {fvar}(T^{\prime })$, they occur unchanged in the core. Thus, the component width of $\mathcal {P}$ is bounded by some constant, and therefore there exist at most polynomial many candidate mappings to be included in *stop*(**S**, **D**). Furthermore, testing each of these mappings is in *FPT*. To see this, recall that testing requires to decide an instance (*∅*, *extcore*(*λ*(*branch*(*t*)),**S**)),*h*, **D** of EXT($\mathcal {C}$). By Theorem 3, this decision is in *FPT* if *extcore*(*∅*, *extcore*(*λ*(*branch*(*t*)),**S**)) = *extcore*(*λ*(*branch*(*t*)),**S**) has bounded treewidth, which is guaranteed by tractability condition (a’).

It thus remains to show the correctness of the algorithm, which follows by the same arguments as in Section 4. Therefore, the only point that needs to be shown is that the presented computation of *stop*(**S**, **D**) is correct. First of all, *stop*(**S**, **D**) according to the definition via the extension core being a subset of *stop*(**S**, **D**) according to the original definition in Section 4 follows immediately. For the opposite direction, where we have already shown that this is not necessarily the case, we show that the additional mappings in *stop*(**S**, **D**) according to the original definition have no effect on the result of Algorithm 1.

Towards this, first assume that for a mapping *ν* ∈*stop*(**S**, **D**) according to the definition in Section 4, there exists an extension $\nu ^{\prime } \colon \lambda (\mathsf {branch}(t)) \rightarrow \mathbf {D}$. Then, since all variables shared between **S** and *λ*(*branch*(*t*)) occur in $\mathcal {I}({\mathbf {S}}, t) $, we have that *ν* can be extended to a homomorphism $\mathbf {S} \rightarrow \mathbf {D}$ if and only if $(\lambda (\mathsf {branch}(t)), \mathbf {S}), \nu ^{\prime }, \mathbf {D}$ is a positive instance of EXT($\mathcal {C}$). I.e., for such *ν* we still have *ν* ∈*stop*(**S**, **D**) by the new definition, and thus the test is correct. Next, assume that there exists no such extension $\nu ^{\prime }$. In this case, in line 7 of the algorithm, $q(\mathbf {D}^{\prime }) = q(\mathbf {D}^{\prime } \setminus \{R_{i}(\nu (\mathbf {v}_{i}))\}$, since *λ*(*branch*(*t*)) is contained in the body of the query, and thus *ν* cannot be part of any solution mapping. Hence, *ν*∉*stop*(**S**, **D**) still gives a correct solution. □

## Relationship with SPARQL and Conclusion

Our results give a fine understanding of the tractable classes of wdPTs in the presence of projection. In particular they show the different sources of hardness. As laid out in the introduction, there is a strong relationship between well-designed SPARQL queries and wdPTs: For every well-designed SPARQL query, an equivalent well-designed pattern tree can be computed in polynomial time, and vice versa, in a completely syntactic way.

Note that our characterization of tractable classes of Theorem 2 unfortunately cannot be immediately translated to well-designed SPARQL queries. This is because our characterization only applies to classes of *simple* well-designed pattern trees. However, RDF triples and SPARQL triple patterns, in the relational model, are usually represented with a single (ternary) relation. Thus, there is no direct translation to and from simple (well-designed) pattern trees. As a consequence, our result does not imply an immediate characterization of the tractable classes of well-designed {AND, OPTIONAL}-SPARQL queries.

Nevertheless, our results also give interesting insights to SPARQL with projections. First, Algorithm 1 directly applies to queries in which relation symbols appear several times and thus in particular for well-designed pattern trees resulting from the translation of well-designed SPARQL queries. Moreover, our result determines completely the tractable classes that can be characterized by analyzing only the underlying graph structure of the queries, i.e., the Gaifman graph. Indeed, since simple queries can simulate all other queries sharing the same Gaifman graph by duplicating relations, Gaifman graph based techniques have exactly the same limits as simple queries. Thus, our work gives significant information on limits of tractability for SPARQL queries in the same way as, e.g., Grohe et al. [13], Chen [4], and Chen and Dalmau [5] did in similar contexts.

As we have seen, by incorporating cores, we can also characterize larger tractable classes in the non-simple case, and thus again for well-designed pattern trees resulting from the translation of well-designed SPARQL queries. However, in this case we do not get a dichotomy result.

Let us mention one major stumbling block towards a characterization of non-simple well-designed pattern trees with projections: In the proof of Lemma 3, we have used a reduction from quantified conjunctive queries. Unfortunately, the tractable classes for the non-simple fragment for that problem are not well understood which limits our result to simple queries since we are using the respective results by Chen and Dalmau [5]. Note that we might have been able to give a more fine-grained result in sorted logics by using the work of Chen and Marx [6], but since this would, in our opinion, not have been very natural in our setting, we did not pursue this direction. Thus, a better understanding of non-simple pattern trees would either need progress on quantified conjunctive queries or a reduction from another problem that is better understood.

One prominent operator of SPARQL that we did not consider is U NION, whose correspondence in pattern trees are sets of pattern trees, so-called pattern forests. While the extension to simple pattern forests is immediate (since no two trees share any relation symbols), it is not clear how to approach the possible repetition of relation symbol within different trees in forests of simple pattern trees in combination with projection.

Finally, another interesting class of queries are weakly well-designed pattern trees. While the tractability conditions can be easily adapted to provide *FPT* algorithms for these queries, providing a characterization of the tractable classes is much harder due to the fact that relevant nodes need not have a descendant introducing a “new” free variable.

## Appendix: Additional Definitions

In Sections 2 and 6, we introduced the notions of restricting a set **A** of atoms to some subset $\mathcal {A} \subseteq \mathsf {dom}(\mathbf {A})$ and projecting a pair of sets of atoms under some mapping. As mentioned in Section 6, below we provide a full formal definition of these notion to avoid any ambiguities a reader may have.

### Restricting a Set of Atoms ($\mathbf {S} \setminus \mathcal {V}$)

A complete definition was already given in Section 2. In the following, we clarify the names of the newly introduced relation symbols *R*^*s*^. For a set **S** of atoms over some relational schema *σ* and $\mathcal {V} \subseteq \mathsf {dom}(\mathbf {S})$, $\mathbf {S} \setminus \mathcal {V}$ contains the following set of atoms over the relational schema $\sigma ^{\prime }$, which is defined implicitly by the newly introduced atoms described below. For every atom $R(a_{1}, \dots , a_{k}) \in {\mathbf {S}}$, let $o_{1}, \dots , o_{\ell }$ be those positions of $(a_{1}, \dots , a_{k})$ that do not contain values from $\mathcal {V}$, i.e., $a_{o_{j}} \notin \mathcal {V}$ for 1 ≤ *j* ≤ *ℓ*, and let $\{i_{1}, \dots , i_{m}\} = \{1, \dots , k\} \setminus \{o_{1}, \dots , o_{\ell }\}$ be those positions such that $a_{i_{j}} \in \mathcal {V}$ for 1 ≤ *j* ≤ *m*. Then the set $\mathbf {S} \setminus \mathcal {V}$ contains the atom $R^{i_{1}=a_{i_{1}}, \dots , i_{m}=a_{i_{m}}}(a_{o_{1}}, \dots , a_{o_{\ell }})$. These are all the atoms in $\mathbf {S} \setminus \mathcal {V}$.

### Projecting a Pair of Sets of Atoms Under a Mapping

Let (**Q**, **D**) be a pair of sets of atoms over the same relational schema *σ* and let *h* be a mapping on (a subset of) *d**o**m*(**Q**). We define the *restriction of* (**Q**, **D**) *under* *h* *as the pair* $({\mathbf {Q}^{\prime }}, \mathbf {D}^{\prime })$ of sets of atoms over the schema $\sigma ^{\prime }$ as follows (the relational schema $\sigma ^{\prime }$ is defined implicitly).

We start by defining $\mathbf {Q}^{\prime }$. For every relation symbol *R* ∈ *σ* and every atom $R(a_{1}, \dots , a_{k}) \in {\mathbf {Q}}$, we distinguish two cases.

- If $\{a_{1}, \dots , a_{k}\} \subseteq \mathsf {dom}(h)$ and $R(h(a_{1}), \dots , h(a_{k})) \in {\mathbf {D}}$, then just ignore $R(a_{1}, \dots , a_{k})$ (i.e., no atom derived from $R(a_{1}, \dots , a_{k})$ occurs in $\mathbf {Q}^{\prime }$).
- Otherwise, let $\{i_{1}, \dots , i_{\ell }\} \subseteq \{a_{1}, \dots , a_{k}\}$ be those positions of $(a_{1}, \dots , a_{k})$ such that $a_{i_{j}} \in \mathsf {dom}(h)$ for 1 ≤ *j* ≤ *ℓ*, and $\{o_{1}, \dots , o_{m}\} = \{1, \dots , k\} \setminus \{i_{1}, \dots , i_{\ell }\}$ those positions such that $a_{o_{j}} \notin \mathsf {dom}(h)$. Then $\mathbf {Q}^{\prime }$ contains the atom $R^{i_{1}=h(a_{i_{1}}), \dots , i_{\ell }=h(a_{i_{\ell }})}(a_{o_{1}}, \dots , a_{o_{m}})$.

These are all atoms in $\mathbf {Q}^{\prime }$.

Observe that the second case includes the possibility that *ℓ* = *k*, i.e., that *a*_*i*_ ∈*d**o**m*(*h*) for all 1 ≤ *i* ≤ *k*, but that $R(h(a_{1}), \dots , h(a_{k})) \notin \mathbf {D}$. In this case, the result is a 0-ary relation symbol $R^{1=h(a_{1}), \dots , k=h(a_{k})}$, and $R^{1=h(a_{1}), \dots , k=h(a_{k})}() \in {\mathbf {Q}^{\prime }}$.

Next we define $\mathbf {D}^{\prime }$. For every relation symbol $R^{i_{1}=b_{1},\dots ,i_{\ell }=b_{\ell }} \in \sigma ^{\prime }$ introduced by the definition of $\mathbf {Q}^{\prime }$ and every atom $R^{i_{1}=b_{1},\dots ,i_{\ell }=b_{\ell }}(a_{1}, \dots , a_{m}) \in {\mathbf {Q}^{\prime }}$, let *k* be the arity of the original relation symbol *R* from *σ*. Then the positions $1, \dots , m$ in $R^{i_{1}=b_{1},\dots ,i_{\ell }=b_{\ell }}$ correspond to positions $j_{1}, \dots , j_{m}$ in *R*. In fact, $\{j_{1}, \dots , j_{m}\} = \{1, \dots , k\} \setminus \{i_{1}, \dots , i_{\ell }\}$. For each atom *R*(**a**) ∈**D** with $a_{i_{r}} = b_{r}$ for all 1 ≤ *r* ≤ *ℓ*, the set $\mathbf {D}^{\prime }$ contains an atom $R^{i_{1}=b_{1},\dots ,i_{\ell }=b_{\ell }}(a_{j_{1}}, \dots , a_{j_{m}})$.

We need to take care of one special case: Assume that, for a pair (**Q**, **D**) and a homomorphism *h* such that **Q** is already a projection of some set **L** of atoms under a set $V \subseteq \mathsf {dom}(\mathbf {L})$, we want to get the projection of (**Q**, **D**) under *h*. I.e. the schema of **Q** already contains relation symbols of the form $R^{i_{1} = b_{1}, \dots , i_{\ell }=b_{\ell }}$. If for any of the *i*_*j*_ (1 ≤ *j* ≤ *ℓ*) it is the case that *b*_*j*_ ∈*d**o**m*(*h*), then in the resulting schema we replace *b*_*j*_ in the name of the resulting atom by *h*(*b*_*j*_). In certain situations, this renaming of the relation symbols will ensure the resulting sets of atoms to be over the same relational schema, which is a prerequisite for finding homomorphisms.

## Footnotes

1 While not stated explicitly by Letelier et al. [17], it is an immediate consequence of their Theorem 3.21.
